# Hybrid Nanomaterials for Cancer Immunotherapy

**DOI:** 10.1002/advs.202204932

**Published:** 2022-12-25

**Authors:** Jianing Li, Wanyue Lu, Yannan Yang, Ruiqing Xiang, Yun Ling, Chengzhong Yu, Yaming Zhou

**Affiliations:** ^1^ Shanghai Key Laboratory of Molecular Catalysis and Innovative Materials Department of Chemistry Fudan University Shanghai 200433 China; ^2^ Institute of Optoelectronics Fudan University Shanghai 200433 China; ^3^ Australian Institute for Bioengineering and Nanotechnology The University of Queensland St Lucia Brisbane 4072 Australia

**Keywords:** cancer immunotherapy, combinational therapy, hybrid nanomaterials, immunoadjuvant, vaccine

## Abstract

Nano‐immunotherapy has been recognized as a highly promising strategy for cancer treatment in recent decades, which combines nanotechnology and immunotherapy to combat against tumors. Hybrid nanomaterials consisting of at least two constituents with distinct compositions and properties, usually organic and inorganic, have been engineered with integrated functions and enormous potential in boosting cancer immunotherapy. This review provides a summary of hybrid nanomaterials reported for cancer immunotherapy, including nanoscale metal–organic frameworks, metal–phenolic networks, mesoporous organosilica nanoparticles, metallofullerene nanomaterials, polymer–lipid, and biomacromolecule‐based hybrid nanomaterials. The combination of immunotherapy with chemotherapy, chemodynamic therapy, radiotherapy, radiodynamic therapy, photothermal therapy, photodynamic therapy, and sonodynamic therapy based on hybrid nanomaterials is also discussed. Finally, the current challenges and the prospects for designing hybrid nanomaterials and their application in cancer immunotherapy are outlined.

## Graphical abstract

The application of hybrid nanomaterials, including metal–organic frameworks, metal–phenolic networks, mesoporous organosilica nanoparticles, metallofullerene, polymer–lipid, and biomacromolecule‐based hybrid nanomaterials, in cancer immunotherapy is summarized. In particular, hybrid‐nanomaterial‐mediated vaccine delivery and combinational immunotherapeutic strategies with, e.g., chemo‐, radio‐, and photo‐, are discussed. The perspectives on the challenges and possible future directions are provided at the end.

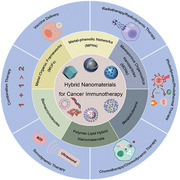

## 1 Introduction

Cancer, characterized by uncontrolled growth and expansion of abnormal cells, is the main leading cause of death worldwide, with ≈19.3 million new cancer cases and almost 10.0 million cancer deaths occurred worldwide in 2020.^[^ ^1^ ^]^ Whereas surgery, chemotherapy, and radiotherapy (RT) are still the frontline approaches for cancer treatment, there are numerous cases in which these treatments caused severe adverse effects on healthy tissues, and/or fail to inhibit tumor recurrence or metastasis.^[^ ^2^ ^]^ In this context, cancer immunotherapy has emerged as a highly promising strategy for the treatment of certain types of cancers, particularly for eradicating metastatic tumors, which is based on the stimulation or recovery of innate and adaptive immunity to recognize and eliminate tumor cells. Since cancer immunotherapy utilizes body's immune system to combat against tumors, its potential adverse effects, although still cannot be completely avoided,^[^ ^3^ ^]^ are dramatically reduced compared to conventional treatments. Unfortunately, only a small portion of patients are benefited from cancer immunotherapy, thus posing a great challenge of improving the therapeutic efficacy and response rate.^[^ ^4^ ^]^

Nanomaterials in combination with immunotherapies have offered a unique solution to address the aforementioned challenge. Thanks to the great efforts devoted in recent years, nanomaterials have shown remarkable promise in promoting cancer immunotherapy in a range of areas, such as cancer vaccine, immunological checkpoint inhibitors, molecular adjuvants, and modulation of tumor microenvironment (TME), leading to significantly improved therapeutic efficacy as well as biosafety.^[^ ^5^, ^6^, ^7^ ^]^ Organic nanomaterials, such as polymers and lipids, are the most classic family of biomaterials applied in drug delivery owing to their excellent biodegradability and biocompatibility.^[^ ^8^, ^9^ ^]^ To date, the majority of U.S. Food and Drug Administration (U.S. FDA)‐approved nanoformulations are based on organic nanomaterials.^[^ ^10^, ^11^ ^]^ This reality catalyzed the research enthusiasm in exploring novel organic nanomaterials, such as protein‐ and nucleic‐acid‐based nanomaterials that emerged recently,^[^ ^12^, ^13^ ^]^ and has achieved encouraging preclinic and clinic outcomes in cancer immunotherapy.

Inorganic nanomaterials, such as ceramics,^[^ ^14^ ^]^ metal‐based materials,^[^ ^15^, ^16^ ^]^ carbon‐based materials,^[^ ^17^ ^]^ and clay materials,^[^ ^18^ ^]^ are also highly appealing materials due primarily to their mechanical, thermal, catalytic, optical, and magnetic properties, tunable porous structure, and consequently diverse functionalities.^[^ ^19^ ^]^ These have laid the foundation for researchers to explore innovative nanotherapeutics systems for immunomodulation and immunotherapy against cancer. However, the biocompatibility of inorganic materials has been a major concern, which, at least in part, explains their limited success in clinic translation.^[^ ^20^ ^]^ This concern is literally reasonable, since our body is mainly made of organic matters that tend to have a broad range of complicated reactions upon interacting with inorganic materials.^[^ ^21^ ^]^

Hybrid materials are defined as the composites consisting of at least two constituents, usually organic and inorganic, at the nanometer or molecular level, which has had a surprisingly long history since the emergence of paints made from inorganic and organic components that were used thousands of years ago.^[^ ^22^ ^]^ However, hybrid nanomaterials have just entered into biomedical use in recent decades.^[^ ^19^ ^]^ They can potentially be engineered to integrate the advantages of biocompatibility and biofunctionality endowed by organic and inorganic components, and/or reveal new properties as a result of hybridization.^[^ ^23^ ^]^ These unique and unprecedented advantages provide the rationale for the extensive research in the field of hybrid nanomaterials, enabling cancer immunotherapy in recent years. A large number of studies have been carried out to construct novel hybrid nanomaterials for boosting the immunotherapeutic outcome, understand their regulatory activity toward immune systems and TME, and create combinatorial or synergistic effects of the material constituents, leading to a series of substantial and exciting progress despite the infancy of this field.^[^ ^24^ ^]^ Notably, several excellent review articles have discussed the biomedical application of hybrid nanomaterials,^[^ ^19^, ^25^ ^]^ but a comprehensive review that specifically focuses on their recent progress in cancer immunotherapy is still lacking to our knowledge.

In this review, we systematically summarize recent research advances on hybrid nanomaterials for cancer immunotherapy. The hybrid nanomaterials are categorized based on their types and characteristics, and their combination with other treatments is also highlighted and discussed. First, we provide an at‐a‐glance introduction on hybrid nanomaterials, including their synthesis, functionality, and biocompatibility. Next, we discuss the organic/inorganic hybrid nanomaterials (e.g., nanoscale metal–organic frameworks (nMOFs), metal–phenolic networks (MPNs), mesoporous organosilica nanoparticles (MONs), and metallofullerene nanomaterials) and their applications in vaccine delivery and combinational immunotherapy, including chemotherapy, chemodynamic therapy (CDT), RT, radiodynamic therapy (RDT), photothermal therapy (PTT), photodynamic therapy (PDT), sonodynamic therapy (SDT), and so on. Other types of hybrid nanomaterials including polymer–lipid and biomacromolecules (e.g., membranes, proteins, and nucleic acids) hybrid nanomaterials for cancer immunotherapy are also discussed. Finally, a summary and outlook is given on the prospects and developments of hybrid nanoparticles for cancer immunotherapy.

## 2 Cancer Immunotherapy

Cancer immunotherapy is considered as one of the most promising treatment modalities owing to its greatly reduced adverse effects compared to current first‐line treatments, including chemotherapy and RT, as well as excellent therapeutic efficacy. Cancer immunotherapy relies on the activation of the immune system to target and eradicate cancer cells, which may result in durable antitumor responses and prevent metastasis and recurrence. In particular, immune checkpoint blockade (ICB), chimeric antigen receptor (CAR) T cell and vaccines have achieved remarkable achievements.^[^ ^26^ ^]^ Cancer immunotherapy has been developed rapidly since the monoclonal antibody ipilimumab was approved. Ipilimumab activates the immune system by targeting cytotoxic T‐lymphocyte‐associated protein 4 (CTLA4), which is the protein expressed on regulatory T cells (Tregs).^[^ ^2^ ^]^ Moreover, other ICBs have been developed using antibodies against programmed cell death‐1 (PD‐1), and programmed cell death ligand‐1 (PD‐L1), which were also approved for melanoma, non‐small‐cell lung cancer, renal cancer, and bladder cancer treatment.^[^ ^27^ ^]^ Therefore, immunotherapy has shown tremendous potential in cancer therapy and various immunotherapeutics are in active development in recent years.

The cancer‐immunity cycle is important mechanism of action for cancer immunotherapy. In general, the cancer‐immunity cycle involves several steps to initiate an effective T‐cell‐mediated immune response, including antigen release from the necrotic or apoptotic tumor cells, antigen uptake by antigen‐presenting cells (APCs) and presentation on major histocompatibility complex class (MHC), antigen presentation to naïve T cells in the draining lymph node, activated tumor‐specific cytotoxic T lymphocytes (CTLs) infiltrate the local tumor site, effector T cells kill cancer cells, and the release of additional cancer antigens.^[^ ^28^ ^]^ Only when each step of the cycle is activated, an effective immune response can be induced. However, M2‐polarized macrophages, myeloid‐derived suppressor cells (MDSCs), Tregs, and immune‐suppressive molecules on tumor cells and T cells enable the tumor to evade the anticancer immune response. Therefore, tumor immune microenvironment needs to be carefully reshaped to improve the outcome of immune therapy. Generally, cancer immunotherapy can be classified as systemic treatment (e.g., vaccines and ICB) and local treatment (e.g., in situ vaccinations), which are usually combined with other traditional therapies to overcome their intrinsic limitations.^[^ ^2^ ^]^

## 3 Hybrid Nanomaterials at a Glance

In this section, we will briefly discuss the fundamentals of hybrid nanomaterials for biomedical applications, which are categorized into three aspects: 1) preparation and surface modification, 2) functionality, and 3) biodegradability and biocompatibility.

### 3.1 Preparation and Surface Modification

In recent years, a wide range of synthetic approaches have been developed to prepare organic/inorganic hybrid nanomaterials, and surface modifications are performed to obtain the desired physicochemical properties for biomedical applications. nMOF nanoparticles are primarily synthesized by the methods including nanoprecipitation, solvothermal, reverse microemulsion, and surfactant‐templated solvothermal reactions.^[^ ^29^ ^]^ However, many of the nMOFs tend to aggregate in aqueous media, so their colloidal stability could be improved by surface modifications (e.g., silica^[^ ^30^ ^]^ and biocompatible polymers^[^ ^31^ ^]^) to increase their performance in biological applications.^[^ ^25^ ^]^ MOFs composed of phenolic ligands with metal ions also fall into the class of MPNs,^[^ ^32^ ^]^ and the fabrication of MPNs is usually more simple, rapid, and environment‐friendly. MPNs can be assembled in one‐step owing to the abundant phenolic groups in polyphenols that can easily coordinate with metal ions. The fabrication process can be completed simply in a basic aqueous condition, which may facilitate the deprotonation of hydroxyl groups to chelate with metal ions.^[^ ^33^ ^]^

In contrast to MOFs and MPNs, the preparation of MONs with various organic groups is complex, which mostly requires surfactant‐directing sol–gel chemistry. The synthesis process of MONs is similar to that of mesoporous silica nanoparticles (MSNs) except for using bissilylated organoalkoxysilane precursors, which can hydrolyze to form silanol groups under the catalysis of structural‐directing agents and alkaline catalysts, then further produce organic R groups‐bridged —Si—R—Si— framework including simple organic bridges (e.g., methylene, ethylene, ethenylene, phenylene, etc.) or relatively complex ones.^[^ ^34^ ^]^

Metallofullerene is synthesized from metal atoms or metal clusters embedding inside fullerenes by a facile solid–liquid method,^[^ ^35^ ^]^ and then modified by functional groups through alkaline reaction,^[^ ^36^ ^]^ which exhibited high potential in biomedicine. The synthesis of polymer–lipid hybrid nanomaterials and biomacromolecules is generally simple. Polymer–lipid hybrid nanomaterials consisting of polymeric core coated with lipid shell are synthesized by coincubation,^[^ ^37^ ^]^ thin‐film hydration,^[^ ^38^ ^]^ and so on. Biomacromolecules, especially hybrid membranes, are usually fused via simple sonication and physical extrusion.^[^ ^39^, ^40^ ^]^ Moreover, biomacromolecules can be utilized as soft templates to prepare hybrid nanomaterials through a biomineralization process.^[^ ^41^ ^]^

### 3.2 Functionality

The unique structures of organic/inorganic hybrid nanomaterials endow them with diverse properties and functions. First, multiple biofunctions can be incorporated into nMOFs and MPNs owning to the intrinsic biological activities of metal ions and organic linkers. For example, some metal ions or organic ligands in nMOFs and MPNs can enhance the generation of reactive oxygen species (ROS) by PDT, RT, RDT, and CDT as nanosensitizers to elicit antitumor effects and promote immunotherapy, and the incorporation of paramagnetic metal ions (e.g., Gd^3+^ and Mn^2+^) can be used for magnetic resonance imaging.^[^ ^42^ ^]^ The excellent adhesion capability of polyphenols allows MPN coating on various interfaces regardless of the structures and shapes, and some polyphenols can also act as both self‐carrier and chemical drug. The coordination between phenolic ligands and metal ions is pH‐responsive or redox‐responsive, which allows the controlled disassembly of MPNs and drug delivery in the acidic tumor area.^[^ ^33^ ^]^ Second, the exceptionally high surface areas with large pore sizes of nMOFs and MONs are conducive to improve cargo loading efficiency, including small‐molecule drugs, nucleic acids, and antibodies.^[^ ^43^, ^44^ ^]^ Notably, MONs could achieve specific drug delivery release, bioimaging, and synergistic therapy in the tumor area rather than normal tissues through the TME‐responsive organic groups within the framework.^[^ ^34^ ^]^ Metallofullerenes especially for gadolinium‐containing metallofullerenes are a new magnetic resonance imaging (MRI) contrast‐enhancing agent. Functionalized metallofullerenes have shown antitumor effects, which may be associated with the modulation of oxidative stress, antiangiogenesis, and immunostimulatory activity.^[^ ^45^ ^]^ The polymer–lipid hybrid nanoparticles have been developed as an effective drug or subunit vaccine delivery platform, which exhibit high stability, favorable encapsulation of hydrophobic drugs, and controlled release attributed to the polymer core.^[^ ^38^ ^]^ Hybrid biomacromolecules including membranes, proteins, and nucleic acids can endow more multifunctional and complex functions of different materials.

### 3.3 Biodegradability and Biocompatibility

As a result of labile metal ligand bonds and nanosize, nMOFs have exhibited excellent biodegradability, which ensures great suitability in the biomedical field.^[^ ^29^ ^]^ However, the relatively large size of nMOFs will limit the enhanced permeability and retention (EPR) effect and the effectiveness needs to be further evaluated.^[^ ^46^ ^]^ The low toxicity and reversible assembly/disassembly characteristics of MPNs make them highly promising for biological application.^[^ ^32^ ^]^ MSNs have demonstrated important value in controlled drug delivery, but the nondegradability of inorganic MSNs stemming from the inert Si—O—Si framework may cause trouble for their excretion and long‐term biosafety in vivo,^[^ ^47^ ^]^ while the incorporation of organic groups, such as disulfide or tetrasulfide bonds, into the framework of MONs can promote their degradation under physiological conditions.^[^ ^34^ ^]^ The metal ions of metallofullerenes are encapsulated in the fullerene cage, which preserves the properties of the metal ion and avoids leakage and thus prevents its dissociation in vivo.^[^ ^45^ ^]^ However, the distribution of metallofullerenes within the body is influenced by the size, modifications, and administration pathways. It is necessary to evaluate the toxicity of fullerenes before their clinical application.^[^ ^45^ ^]^ Lipid‐based nanoparticles have been widely used owing to their good biocompatibility and convenient modification of the surface, but are limited by the poor drug‐loading ability and rapid release.^[^ ^48^ ^]^ Hybrid lipid‐shell polymer‐core nanoparticles can improve the stability, reduce cytotoxicity, and decrease leakage of the encapsulated drugs owned to the lipid layer.^[^ ^49^ ^]^ Hybrid biomacromolecules inherently possess high biocompatibility, thus have been widely recognized as ideal candidates for drug delivery. Currently, enormous efforts have been devoted to improving the functionality of hybrid biomacromolecules, which is expected to further benefit their clinic use.

## 4 nMOFs for Cancer Immunotherapy

The integration of nanotechnology with cancer immunotherapy is a promising development direction. Nanotechnology provides a promising strategy to enhance the efficacy of various combinational immunotherapies to potentiate cancer immunotherapy. Nanoparticles can serve as carriers for antigens, immune adjuvants, immune agonists, and chemotherapeutic drugs, protecting them from degradation and prolonging their survival in the body.^[^ ^50^, ^51^ ^]^ Nanoparticles can passively accumulate at tumor sites through EPR effect, or they can actively target lymph nodes, antigen‐presenting cells, or tumor cells through surface modification and size adjustment to reduce the toxic and side effects of exogenous drugs.^[^ ^50^ ^]^ Metallic nanoparticles like gold nanoparticles (NPs) and iron oxide NPs even provide in situ imaging facilities.^[^ ^52^, ^53^ ^]^

Nanoparticles such as liposomes, metal nanoparticles, polymers, mesoporous silica, carbon nanotubes, calcium phosphate NPs, and nMOFs have been used to construct nanocarriers.^[^ ^31^, ^54^ ^]^ Owing to the large surface areas, highly ordered porosity, and well‐defined structures, nMOFs will have promising ability of loading and releasing for therapeutic agents. In consideration of biocompatibility, the most often studied nMOFs are Materials of the Institute Lavoisier (MIL),^[^ ^55^ ^]^ zeolitic imidazolate frameworks (ZIFs),^[^ ^56^ ^]^ porous coordination networks (PCNs),^[^ ^57^ ^]^ and University of Oslo nanoparticles.^[^ ^58^, ^59^ ^]^

Among many nanoformulations, nMOFs have emerged as a unique class of inorganic–organic hybrid nanomaterials with several favorable attributes for biological applications owing to excellent biocompatibility, biodegradability, suitable size, ease of modification, and functionalization. Furthermore, the water solubility of nMOFs and improvement of hydrophobicity for drugs endow MOF‐based nanoparticles with the potential of becoming promising drug‐loading platforms for next‐generation targeted nanomedicines. Significantly, they have also been used as photothermal platforms^[^ ^60^ ^]^ and photosensitizers^[^ ^61^ ^]^ to enhance the efficacy of treatments further. Compared with other nanomaterials, the nature of nMOFs such as metal ions, ligands, crystallinity, and porosity gives nMOFs unique advantages. As components of the secondary building unit (SBU), metal ions and ligands can work as sources of functionalization. For example, some metal ions trigger Fenton or Fenton‐like reactions in vivo to generate reactive oxygen species and then cause intracellular oxidative stress,^[^ ^62^ ^]^ which enables nMOFs to be utilized as nanomedicines for CDT. Other functional metal ions like aluminum, which work as an immune adjuvant, can be integrated into nMOFs enhancing the immune responses against cancers.^[^ ^63^ ^]^ On the other hand, some functional ligands such as porphyrins and their derivatives can also impart photosensitive properties to nMOFs, further broadening the range of applications for MOFs to photosensitizers.^[^ ^64^ ^]^ Distinguished from other nanomaterials, the simplicity and diversity of nMOF synthesis enhance the ability of drug loading of nMOFs. The encapsulation and adsorption of protein, drugs, or multiple classes of bioactive molecules can be achieved easily owing to the porous structure,^[^ ^65^ ^]^ and immune adjuvants can also be linked to organic ligands through hydrophobic interactions. Biomineralization via a self‐assembly process is an efficient way to encapsulate protein. This strategy has been widely applied to provide biomacromolecules exoskeletal‐like protection for immune shielding and preservation of bioactivity.^[^ ^66^ ^]^

This section focuses on recent development of MOFs as new tools combining immunotherapy with chemotherapy, PDT, CDT, PTT, RT, and RDT. It is worth mentioning that nMOFs sometimes do not activate immunity directly and only act as drug carriers. As a porous material, the high porosity and specific surface area provide a high drug load. The modifiability of organic ligands makes the modification more available, and eventually achieves the functions of targeted drug delivery, increased cellular uptake, and controlled drug release. We consider this indirect effect important in immunotherapy as well.^[^ ^67^, ^68^ ^]^

### 4.1 nMOFs as Nanocarrier for Vaccines, Adjuvants, and Drugs

In the past few decades, immunotherapy has received increasing attention as a promising treatment for cancer.^[^ ^69^ ^]^ Cancer immunotherapy is based on the presence of tumor antigens on the surface of tumor cells that can be recognized by the immune system, thereby activating the antitumor immune system.^[^ ^70^ ^]^ Among several cancer immunotherapies, the cancer vaccine is a commonly used strategy. Compared to directly isolating antigens and constructing tumor vaccines in vitro, in situ generation of tumor vaccines in vivo has achieved more success, partly due to improved immunogenicity.^[^ ^71^ ^]^ In situ tumor vaccines will induce cancer cell death, promote tumor antigen release, further enhance the maturation of APCs, and induce antitumor immune responses. Light, X‐rays, and certain chemotherapy drugs can be the keys to triggering in situ cancer vaccines.^[^ ^72^ ^]^

As nanoplatforms for drug delivery and cancer immunotherapy, nMOFs, a series of hybrid materials, have unique advantages: first, the synthesis and postmodification of nMOFs are convenient, and their size and morphology can be controlled, thus the nanoparticles have excellent targeting properties. Second, the porous structure and high porosity of nMOFs significantly improve the loading capacity of proteins and drug molecules, giving access to the potential combination of chemotherapy and immunotherapy. It is worth noting that specific nMOFs can achieve tumor‐microenvironment‐responsive degradation to control drug release and reduce drug toxicity. For example, the coordination bond between Zn^2+^ and 2‐methylimidazole in zeolitic imidazolate framework‐8 (ZIF‐8) is unstable under acidic conditions, enabling pH‐responsive degradation in the tumor microenvironment. **Table** **1** summarizes the reported nMOFs used for vaccines or adjuvant delivery.

**Table 1:** Various types of nMOFs used for vaccine or adjuvant delivery

MOF	Target	Stimuli‐responsive cargo release	Payload and encapsulation	Outcomes/results	Ref.
ZIF‐8	Antigen presenting cells (APCs)	pH‐responsive	Cytosine–phosphate–guanine (CpG) and ovalbumin (OVA)	Induction of a potent immune memory response, and strong humoral and cellular immunity in vitro and in vivo	[73]
Uio‐66 (biomineralized by calcium phosphate)	APC	pH‐responsive degradation of (CaP) exoskeleton and PO_4_^3−^‐responsive DNA release	CpG	Stimulation of potent immunostimulation in living macrophage cells and upregulation in the stimulated secretion of cytokines (IL‐6, TNF‐*α*)	[74]
Eu3+–GMP	Tumor cells	pH‐responsive	CpG and OVA	Enhancement of antigen cross‐presentation and the recruitment of tumor‐killing immunocytes (CD8+ T cells and NK T cells)	[75]
ZIF‐8	Tumor cells (cancer cell membrane coating)	pH‐responsive	catalase (CAT) and doxorubicin (DOX)	Combination of chemotherapy and immunotherapy (anti‐PD‐L1), downregulate the expression of hypoxia‐inducible factor 1*α* and programmed death ligand 1 (PD‐L1)	[76]
ZIF‐8	Tumor cells	pH‐responsive	DOX	Combination of chemotherapy and avasimibe immunotherapy, enhancement of cytotoxic T lymphocytes (CTL) infiltration in tumors	[77]
ZIF‐8 (biomineralized by Al^3+^)	Lymph nodes	pH‐responsive	CpG and OVA	Enhancement of antigen cross‐presentation and induction of strong antigen‐specific humoral and CTL responses with minimal cytotoxicity	[63]
Uio‐66	Bone cells (zoledronic acid (ZOL) modification)	–	CpG	Suppression of osteoclast‐mediated bone destruction and enhancement of polarization of tumor‐resident macrophages to M1 phenotype	[78]
MIL‐88A	–	pH‐responsive	Minicircle DNA(MC) encoding anti‐CD3/anti‐EpCAM‐bispecific T‐cell engager (MC.BiTE)	High in vivo expression product of the BiTE‐induced T‐cell‐mediated cytotoxicity against human ovarian cancer SKOV3 cells	[79]
Al^3+^, Ru^3+^, and 2‐aminoterephthalic acid	APC	PO4^3−^‐responsive Al^3+^ release	–	PTT (Ru) cooperates with immune adjuvant (Al) and anti‐PD‐L1 to recruit and activate APC, stimulate T‐cell proliferation and activation	[75]
ZIF‐8	Tumor cells (cancer cell membrane coating)	pH‐responsive	Nivolumab (NV)	Reducing immune‐related toxicity and increasing patient compliance of ICB and enhancement of antitumor activity due to the preferential accumulation and prolonged retention of NV	[80]
Eu^3+^–GMP	APC	pH‐responsive	OVA (loaded by MSN) and CpG	Induction of innate immunity and adaptive immune system to be biased toward the Th1‐type cellular immune response	[81]
ZIF‐8 (biomineralized by CaCO_3_)	APC (lysosome‐targeting aptamer)	pH‐responsive	Perforin and granzyme B	Perforin, granzyme B, and Ca^2+^ reprogrammed CD8^+^ T Cells to enhance the insufficient targeting of T cells to the tumor area	[82]
ZIF‐8	Tumor cells	pH‐responsive	g‐C_3_N_4_–Au, CO_2_	Combined with CO gas therapy, a light‐controllable release behavior of CO, which gradually aggravates the oxidative stress in tumor cells to induce ICD	[83]
ZIF‐8	Tumor cells	pH‐responsive	Mitoxantrone (MIT) and hydralazine (HYD)	Introduction of an apoptosis‐to‐pyroptosis transformation with a potential disruption of MDSC‐mediated T‐cell paralysis	[84]
Uio‐66	Tumor cells	PO_4_^3−^‐responsive	NLG919 (IDO inhibitor) and chlorambucil‐based prodrug (CLB)	Inhibition of IDO activity by NLG919 reverses the immunosuppressive tumor microenvironment, chemotherapy drugs are precisely activated in the presence of near‐infrared light, triggering immunogenic cell death (ICD)	[85]
MOF‐5 (doped by Gd^3+^)	Tumor cells	pH‐responsive	–	Intracellular Zn^2+^ overload activates endoplasmic reticulum stress for ICD induction, Gd^3+^ modulates the cell signaling and immunosuppressive microenvironment	[86]
ZIF‐8	Tumor cells	pH‐responsive	Gemcitabine (Gem) and d‐1‐methyltryptophan (d‐1‐MT)	NPs efficiently decrease OS cell viability and reactivate antitumor immunity by inhibiting indoleamine 2,3 dioxygenase and myeloid‐derived suppressor cells	[87]
Zr^4+^–Fe–TCPP	M2‐like tumor‐associated macrophages (TAM)	–	Diclofenac (Dic)	Codelivery of Fe and Dic decreases the efflux by hepcidin/ferroportin signaling pathway, enabling enhanced intracellular accumulation for improved M2‐to‐M1 macrophage repolarization	[88]
ZIF‐8	Tumor cells	pH‐responsive	curcumin (CUR) and BMS1166	BMS1166 inhibits the interaction between PD‐1 and PD‐L1 and CUR induces autophagy to increase ICD	[89]
ZIF‐8	Tumor cells	pH‐responsive	Tumor cells	The whole‐cell cancer vaccines (WCCVs) with WCCV‐in‐shell structure with enhanced immunogenicity ascribing from the surface‐exposed calreticulin to promote dendritic cell recruitment, antigen presentation, and T‐cell activation	[90]
ZIF‐8	Bone marrow dendritic cells (BMDCs)	pH‐responsive	OVA and polyinosinic–polycytidylic acid (polyIC)	In combination with systemic checkpoint blockade at merely 10% dose of PD‐1 blockade monotherapy, exhibits synergetic effects that reverse the immunosuppressive tumor microenvironment and activate strong immune reaction	[91]

#### 4.1.1 nMOFs for Vaccine Delivery

nMOFs can codeliver antigen and adjuvant and release them simultaneously in APCs, and the colocalization of antigen and adjuvant can activate a more effective immune response.^[^ ^63^, ^73^, ^81^ ^]^ Zhang et al. first designed the MOF‐based vaccines by encapsulating ovalbumin (OVA) and attaching the cytosine–phosphate–guanine oligodeoxynucleotides (CpG ODNs) in/on ZIF‐8 nanoparticles. This nanoplatform can induce efficient humoral and cellular immunity and generate immune memory.^[^ ^73^ ^]^ Duan et al. loaded OVA into complexes of Eu^3+^ and guanine monophosphate (GMP) in one‐pot coprecipitation and the introduced CpG through Watson–Crick base pairing, which could increase CpG loading. Appropriately sized nMOFs enhance macrophage uptake and protect antigens from clearance in vivo. The pH‐responsive drug release can induce strong cellular immune responses for cancer therapy.^[^ ^75^ ^]^ Zhong et al. designed a natural biomineralization process to create aluminum‐containing ZIF‐8 particles for OVA and CpG delivery.^[^ ^63^ ^]^ Al^3+^ could work as an adsorbent that enhances the phagocytosis of antigens by APC to augment antigen‐specific CD8^+^ T‐cell responses and promote Th1 type immunity. Cross‐presentation is a process that CD8a^+^ dendritic cells (DCs) and CD103^+^ DCs can specifically present exogenous antigens on MHC‐I molecules, thereby priming CTLs.^[^ ^92^ ^]^ The key to the cross‐presentation involves i) lysosomal proteases cleaving and fragmenting the antigen; ii) the cleaved protein being released into the cytoplasm. After being uptaken by cells, bonds of zinc ions and imidazole of ZIF‐8 could break within the lysosome, causing lysosome swelling and rupture due to protonation. OVA encapsulated in pH‐responsive ZIF‐8 based nanoparticles (ZNPs) and Al^3+^ dopped ZNPs (ZANPs) can escape from lysosomes, release into the cytoplasm, and induce cross‐presentation eventually, which is essential for the initiation of CD8^+^ T‐cell responses for cancer immunotherapy. Then, antigens can be cross‐presented as demonstrated by the expression of H‐2Kb—SIINFEKL. Interestingly, the doping of Al^3+^ enhanced antigen cross‐presentation. Attaching CpG to nanoparticles further increased the proportion of H‐2Kb—SIINFEKL‐positive bone marrow dendritic cells (BMDCs).

#### 4.1.2 nMOFs for In Situ Vaccination

Compared to the targeted delivery of antigens, in situ vaccination offers neoantigens that are expressed only in the tumor site, which would provide less systemic toxicity and greater immunogenicity.^[^ ^93^ ^]^ The in situ vaccination provides tumor‐specific antigens and induces tumor cell death by chemotherapy, oncolytic virus, etc.; or introduces immune activators (interleukins (IL) like IL‐2, IL‐7, stimulator of interferon gene (STING) agonists, etc.) to activate local innate immunity and adaptability of tumor immunity.

**Figure 1:**
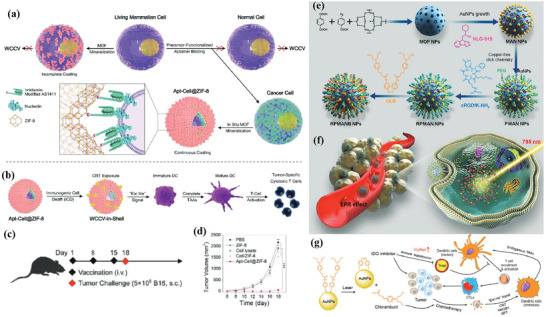
a) Schematic illustration of cell‐selective encapsulation with ZIF‐8 shells via biocompatible aptamer‐based mineralization. b) Mechanism of Apt‐Cell@ZIF‐8 inducing immune responses. c) Schematic of the immunization strategy for tumor prevention. d) The average tumor volumes over time for each group after tumor challenge. Reproduced with permission.^[^ ^90^ ^]^ Copyright 2022, Wiley‐VCH. e) Schematic illustration showing the preparation of the hybrid nanomedicine NPs with the ability to co‐deliver a chemotherapeutic prodrug (CLB) and an IDO inhibitor (NLG919). f) Targeted drug delivery by exploiting the EPR effect and receptor‐mediated endocytosis, followed by the drug release and activation inside the cells. g) Simplified mechanism of RPMANB‐NP‐mediated chemo‐immunotherapy by eliciting tumor immunogenicity and overcoming immunosuppressive tumor microenvironment. Reproduced with permission.^[^ ^85^ ^]^ Copyright 2021, Wiley‐VCH.

Typically, some small molecules and chemodrugs can efficiently induce immunogenic cell death (ICD), such as doxorubicin (DOX), cisplatin, and gas molecules. Xiao et al. reported a photocatalytic nMOF‐based nanogenerator that can integrate gas therapy and immunotherapy.^[^ ^83^ ^]^ The g‐C_3_N_4_–Au nanocomposite is encapsulated into ZIF‐8 as a photocatalyst for catalyzing the reduction of CO_2_ and the high porosity of MOF enhances CO_2_ collection. CO production promotes ICD and generates tumor antigens in situ through ROS production and mitochondrial damage. Combination with ICB (anti‐PD‐L1) can amplify this immune effect, effectively suppressing primary and distal tumor growth. Recently, Yang et al. used MOFs as nanoshells to cover living cells to achieve selective single‐cell encapsulation.^[^ ^90^ ^]^ During the coating process, cancer cells undergo ICD due to changes in cell stiffness, and subsequently large amounts of calreticulin (CRT) are exposed on the surface of the MOF. As shown in **Figure** **1**a,b, the whole‐cell cancer vaccine (WCCV) prepared by this method retains the complete antigen information to the greatest extent, and together with the highly expressed CRT on the surface of NPs, strongly stimulates DC maturation, antigen cross‐presentation, and T‐cell activation. After vaccination, WCCV‐in‐shell shows much better tumor immunoprophylaxis than either the imperfectly coated cancer cells or the traditional WCCV. Although Apt‐Cell@ZIF‐8, the WCCV, could be metabolized by liver after intravenous administration, the antigen carried by Apt‐Cell@ZIF‐8 was efficiently presented to the lymphatic nodes with long‐term retention in draining lymph nodes, which would be beneficial for WCCV‐in‐shell to achieve long‐lasting protective immunity. These vaccinated mice received a further challenge of subcutaneous B16 cells on the right side of the mouse (Figure 1c,d). The results show the enhanced immunogenicity of Apt‐Cell@ZIF‐8, leading to significant reduction of average tumor size of 30.83 mm^3^.

Limited by the immunosuppressive microenvironment, the ICD induced by a single drug is usually insufficient to activate a robust immune response. Therefore, simultaneous drug delivery and modulation of the tumor microenvironment are crucial. The high porosity of nMOFs provides a suitable platform to achieve this goal. Ding et al. improved the immunogenic cell death by delivering and in situ activating chlorambucil‐based prodrugs (CLB) and designed the nanoplatform called RPMANB.^[^ ^85^ ^]^ Notably, CLB can be precisely activated by near‐infrared light through efficient plasmon‐driven catalysis, avoiding toxic side effects. In the synthesis of MOF, the addition of a monocarboxyl molecule (4‐azidobenzoic acid, BCN_3_) was utilized as a modulator to finely tune the size and morphology, and the loading of the indoleamine 2,3‐dioxygenase (IDO) inhibitor (NLG919) (Figure 1e–g). This inhibitor can be released in the TME with high phosphate concentrations and reverses the immunosuppressive TME by inhibiting the activity of IDO enzymes. The chemo‐immunotherapy greatly suppresses tumor growth by promoting the intratumoral accumulation of CTLs and downregulating regulatory T cells. Recently, it has been proved that MOF can integrate the dual functions of inducing ICD and enhancing immune response by itself, without loading functional small molecules. Dai et al. synthesized Gd–MOF‐5 to enhance ICD.^[^ ^86^ ^]^ On the one hand, nMOFs deliver zinc ions to cancer cells, intracellular Zn^2+^ overload causes mitochondria dysfunction, activates endoplasmic reticulum (ER) stress, and disrupts cellular calcium homeostasis, leading to ICD induction. On the other hand, Gd^3+^ competes with Ca^2+^ for transmembrane protein 16F (TMEM 16F) binding, and phosphatidylserine (PS) works as an immunosuppressive signal on the surface of cancer cells to counteract the efficacy of ICD. Therefore Cd^3+^ eventually deactivates TMEM 16F and inhibits PS externalization. When combined with an anti‐PD‐L1, Gd–MOF‐5 activated a potent immune response and effectively inhibited primary and distal tumor growth in a bilateral 4T1 tumor model. Autophagy is another pathway that activates immunogenic death. Ge et al. developed a pH‐responsive nanoplatform with the loading of curcumin (CUR) and BMS1166.^[^ ^89^ ^]^ As a natural derivative, CUR can promote ICD in OS by activating autophagy, and subsequently boost the maturation of DCs. Furthermore, when combined with ICB, by adding the small molecule BMS1166 to inhibit PD‐1/PD‐L1 interaction, NPs induced a strong immune response and remodeled the immunosuppressive microenvironment of OS, promoting tumor infiltration of CTL.

### 4.2 nMOFs for Combinational Immunotherapy with Chemotherapy

Benefiting from the simplicity and diversity of nMOF synthesis, protein encapsulation, and adsorption can be achieved conveniently, and drug molecules can also be linked to organic ligands through hydrophobic interactions. This means that drugs and proteins can be coassembled and specifically released from nMOFs, enabling the combination of chemotherapy and immunotherapy. In this case, nMOFs usually act as drug carriers which can improve the pharmacokinetics of drugs. Zhou et al. developed a nano ZIF‐8 (nZIF‐8) encapsulating mitoxantrone (MIT) and hydralazine (HYD).^[^ ^84^ ^]^ The constructed nanoplatform achieves the shift of cells from immunosuppressive apoptosis to immunostimulatory pyroptosis through the targeted release of MIT, and promotes the antigen recognition of APCs. HYD can prevent the formation of methylglyoxal in MDSCs, thereby dismissing T‐cell paralysis. Disruption of the immunosuppressive microenvironment and establishing an effective immune response ultimately exhibit excellent anticancer and antimetastatic activity. In addition to chemotherapeutic drugs, intracellular iron accumulation also induces pyroptosis, which activates the immune system. Coating MIL‐100(Fe) MOF nanoparticles with liposomes can introduce high amounts of iron ions into cells.^[^ ^94^ ^]^ Fan et al. designed another nZIF‐8‐based nanoplatform with the loading of gemcitabine (Gem) and d‐1‐methyltryptophan (d‐1‐MT).^[^ ^87^ ^]^ The former can effectively reduce osteosarcoma cell viability (proliferation, apoptosis, cell cycle, migration, and invasion), and the latter can reactivate antitumor immunity by inhibiting indoleamine‐2,3‐dioxygenase and myeloid‐derived suppressor cells.

Biomineralization via a self‐assembly process has been widely applied to provide biomacromolecules exoskeletal‐like protection for immune shielding and preservation of bioactivity.^[^ ^66^ ^]^ During self‐assembly (biomineralization), the activity of the biomolecules or living organisms is retained and even improved in some cases.^[^ ^95^ ^]^ Wang et al. reported a Uio‐66‐based nMOF with the loading of CpG, which was further armed by a protective shell of calcium phosphate (CaP) exoskeleton.^[^ ^74^ ^]^ The CaP exoskeleton dissolves in the acidic environment when the nanoparticle reaches endolysosomes and in situ generates free phosphate ions. As a result, CpG is released from nMOFs, stimulates potent immunostimulation in living APCs, and subsequently enhances the cytokine secretion. Zhang and co‐workers used calcium carbonate (CaCO_3_) to induce MOF mineralization, improve the composite material's stability in encapsulating therapeutic protein, and provide calcium ions with synergistic effects.^[^ ^82^ ^]^ Through preloading of perforin and granzyme B, which are T‐cell‐needed therapeutic proteins for tumors, the nanoplatform achieved reprogramming of T cells and stimulated the differentiation of CD8^+^ T cells, improving the effect of T‐cell immunotherapy. The surface modification of lysosome‐targeting aptamer (CD63 aptamer) provides T‐cell lysosome‐targeting properties and ensures efficient protein release.

The combination with ICB is an essential module of MOF immunotherapy. When integrated with chemotherapy, RT, and PDT, MOF‐induced ICD will enhance the therapeutic effect of ICB, which will be introduced in subsequent chapters. This subsection would like to highlight the role of MOFs as nanocarriers in ICB therapy. First, the MOF itself can deliver relevant immune checkpoint inhibitors to directly enhance the therapeutic effect of ICB.^[^ ^80^ ^]^ On the other hand, MOFs can modulate the tumor microenvironment by loading immune adjuvants^[^ ^91^ ^]^ or other small molecules,^[^ ^76^ ^]^ reversing it from an immunosuppressive state to an immune activation state. Alsaiari et al. developed biomimetic ZIFs with cancer cell membrane coating and used them for the controlled delivery of nivolumab (NV), a monoclonal antibody checkpoint inhibitor.^[^ ^80^ ^]^ The sustained release behavior of NV–ZIF has shown a higher efficacy than the naked NV to activate T cells in hematological malignancies. Li and co‐workers designed a universal self‐assembly route to integrate OVA into MOF‐gated mesoporous silica (MS) as a cancer vaccine.^[^ ^81^ ^]^ MS works as the drug carrier here, and MOF acts as a gatekeeper to protect the antigen and realize specific release. Combination of nanoplatforms with systemic PD‐1 blockade therapy produces a synergistic effect, enhances antitumor immunity, and reduces the effective dose of anti‐PD‐1 antibody to as low as 10% for PD‐1 blockade monotherapy, and consequently elicits robust adaptive OVA‐specific CD8^+^ T‐cell responses and generates immune memory. Zou and co‐workers reported a ZIF‐8‐based core–shell nanoplatform for impairing therapeutic effects including chemotherapy and advancing ICB therapy.^[^ ^76^ ^]^ By embedding with catalase and DOX, the nMOFs work as an oxygen generator and drug reservoir that possesses the ability to alleviate tumor hypoxia, which is highly correlated with the expression of PD‐L1. Therefore, combined with ICB therapy with anti‐PD‐1, nMOFs achieved enhanced inhibition of PD‐1/PD‐L1 binding and markedly activated immune responses in antitumor and antimetastasis. Both aspects have played a decisive role.

Besides ICB therapy, the development of antitumor drugs targeting M1 phenotype macrophage polarization is one of the focuses of current immunotherapy. nMOFs can be designed for tumor‐associated macrophage (TAM) targeting and to improve macrophage repolarization from M2 to M1. Wei et al. developed M2‐macrophage‐targeting peptide‐conjugated iron‐based MOFs to load diclofenac (Dic) for enhanced cancer immunotherapy.^[^ ^88^ ^]^ Iron‐based nanoparticles exhibit the ability to repolarize M2 to M1 macrophages, and Dic increases intracellular iron accumulation by reducing the efflux of the hepcidin/ferroportin signaling pathway. Through efficient macrophage repolarization, MOFs remodel the tumor immune microenvironment to generate long‐term antitumor immune memory. Pang et al. designed CpG‐loaded MOF nanoparticles with bone targeting capabilities by surface modification with zoledronic acid (ZOL), FDA‐approved antiresorptive bisphosphonate.^[^ ^78^ ^]^ As previous report, the osteoclasts are formed through the fusion of mononuclear macrophage cells following stimulation with receptor activator of nuclear factor‐*κ* B Ligand (RANKL). Activation of toll‐like receptor 9 (TLR9) by CpG inhibits RANKL‐induced osteoclastogenesis and induces polarization of mononuclear macrophage cells toward a proinflammatory M1 phenotype. Eventually, nanoparticles reduced the deleterious effects of osteolysis and destruction of bone metastases and inhibited tumor growth and progression.

### 4.3 nMOFs for Combinational Immunotherapy with PDT

PDT uses photosensitizers (PSs), oxygen, and light to destroy tumors through direct cell kill, microvascular disruption, and inflammation. Under the induction of light, ROS produced by PSs can kill tumors. Since tumor hypoxia is one of the main problems faced by oxygen‐dependent PDT, the combination with nonoxygen‐dependent therapies such as immunotherapy is a promising strategy.^[^ ^96^ ^]^ Furthermore, PDT has shown the ability to induce antitumor immunity like induction of ICD and release of tumor‐associated antigens (TAAs) during cancer cells' destruction, leading to the activation and proliferation of CD8^+^ T lymphocytes. On the other hand, due to the short life span of ROS, it is important to precisely deliver the drug to the desired area and improve intracellular diffusion. As reported before, most PSs used for PDT are hydrophobic and prone to aggregation and quenching. Therefore, it is important to design suitable and hydrophilic nanoplatforms for the delivery of PSs.

A new class of nanophotosensitizers (nPSs) based on nMOFs has recently emerged as highly effective PSs for PDT.^[^ ^97^, ^98^, ^99^ ^]^ The nMOFs can passively accumulate in tumor tissues via EPR effect, reducing damage to normal tissues. Importantly, nMOF‐based nPSs directly incorporate PSs as the building units, allowing for high PS loadings without self‐quenching^[^ ^100^ ^]^ and the high porosity of nMOFs will also be more favorable for the diffusion of ROS to enhance the effect of PDT. **Table** **2** summarizes the nMOF‐based nPSs used for PDT and immunotherapy.

**Table 2:** The nMOF‐based nPSs used for combinational immunotherapy with PDT

MOF	Metal	Ligand	Biological integration method	Payloads	Application (combined with immunotherapy)	Ref.
Fe–TBP	Fe	5,10,15,20‐tetra(*p*‐benzoato)porphyrin (TBP)	Singlet oxygen generation	–	PDT and CBI	[101]
W–TBP	W	TBP	ROS generation and encapsulation	CpG ODN	PDT, checkpoint blocking immunotherapy (CBI), and immune adjuvants	[102]
PCN–ACF–CpG@HA	Zr	Tris(chlorisopropyl)phosphate (TCPP)	ROS generation and encapsulation	CpG ODN and Acriflavine (ACF)	PDT, immune adjuvants, antihypoxic signaling inhibitor, and targeted cancer treatment	[103]
TPZ encapsulated core–shell upconversion nanoparticle@porphyrinic MOFs (TPZ/UCS)	Zr	Porphyrin	ROS generation and encapsulation	TPZ	NIR‐light‐induced PDT, hypoxia‐activated chemotherapy, and CBI	[104]
Apt/PDGs–s@pMOF	Zr	TCPP	ROS generation and encapsulation	DGL–Gem (PDG)	PDT, targeted cancer treatment, and immune adjuvants	[105]
SNP@ZrMOF	Zr	TCPP	ROS generation and encapsulation	Rose Bengal	PDT	[106]
NaLnF_4_@MOF	Zr, Eu	TCPP	ROS generation	–	PDT, NIR‐II imaging, and CBI	[107]
Cu–TBP	Cu	TBP	ROS generation and pH‐responsive degradation	–	PDT, CDT, and CBI	[108]
Vorinstat (SAHA)‐loaded manganese‐porphyrin metal‐organic framework with modification of AS1411 aptamer (SMTA)	Mn(III)	TCPP	Nuclear DNA damage, chromatin decompaction, cGAS–STING pathway activation, and GSH‐responsive degradation	SAHA	Nucleus‐targeted delivery, PDT, and coactivation of innate and adaptive immunity	[109]
CuTPyP/F68	Cu	TPyP	ROS generation and encapsulation	CUR	Targeted cancer treatment, PDT	[110]
Fe‐proto‐porphyrin‐based hybrid metal–organic frameworks with a surface coated with red blood cells membranes（FTP@RBCM)	Fe(III)	TCPP	ROS generation, GSH‐responsive degradation	Pt NPs	Targeted cancer treatment, PDT, CDT, and CBI	[111]
IDOi@TBC–Hf	Hf	5,10,15,20‐Tetra(*p*‐benzoato)chlorin (TBC)	ROS generation	IDOi	PDT, IDO inhibition	[112]
TBP–MOF	Zr	TBP	ROS generation	–	PDT, CBI	[113]
PCN‐SU	Zr	TCPP (sulfonation)	ROS generation	–	NIR‐light‐boosted PDT, CBI	[114]

#### 4.3.1 nMOFs for Overcoming Tumor Hypoxia

**Figure 2:**
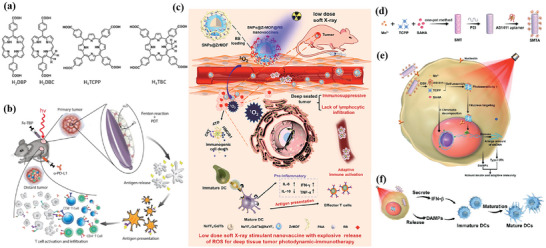
a) Common photoactive ligands for MOF construction. b) Schematic illustration of using Fe–TBP to overcome hypoxia for PDT primed cancer immunotherapy. Reproduced with permission.^[^ ^101^ ^]^ Copyright 2018, American Chemical Society. c) Schematic illustration of the soft‐X‐ray‐responsive nanoprobe with the explosive generation of ROS for deep tissue synergistic PDT–immunotherapy of tumor and modulating the immunosuppressive TME. Reproduced with permission.^[^ ^106^ ^]^ Copyright 2021, Wiley‐VCH. d) Schematic illustration of the synthetic procedure of SMTA. e) Schematic illustration of the proposed mechanism of PIS‐mediated photodynamic immunotherapy. f) Schematic illustration of innate and adaptive immune coactivation by SMTA. Reproduced with permission.^[^ ^109^ ^]^ Copyright 2022, Wiley‐VCH.

In fact, the effect of PDT is limited mainly by the status of hypoxia in tumor cells.^[^ ^96^ ^]^ Therefore, decreasing the oxygen dependence of photosensitizers is a promising approach.^[^ ^101^, ^113^, ^114^ ^]^ Lan et al. designed a nanoscale metal–organic framework, Fe–5,10,15,20‐tetra(*p*‐benzoato)porphyrin (TBP), as a novel nPS to overcome tumor hypoxia and sensitize effective PDT, priming noninflamed tumors for cancer immunotherapy (**Figure** **2**a).^[^ ^101^ ^]^ As shown in Figure 2b, the nanoplatform induces PDT under both normoxic and hypoxic conditions efficiently due to the porphyrin ligands. On the other hand, the iron–oxo cluster endows the nanoparticle with the ability to trigger the Fenton reaction, which catalyzes the decomposition of H_2_O_2_ to generate O_2_ to overcome hypoxia. When combined with anti‐PD‐L1 treatment, the nMOF induces significant expansions of both CD4^+^ and CD8^+^ cytotoxic T cells in a mouse model of colorectal cancer, which infiltrates distant tumors to elicit abscopal effects and results in >90% regression of tumors. To improve the photophysical of nPSs, Zeng et al. reported properties of benzoporphyrin‐based metal–organic framework (TBP–MOF), which exhibited 10‐connected Zr_6_ cluster and much improved photophysical properties due to the *π*‐extended benzoporphyrin‐based linkers.^[^ ^113^ ^]^

#### 4.3.2 nMOFs for Enhancement of PDT Efficiency

Another shortage faced by MOF‐based PDTs is that most of them work mainly by visible light, which severely limits the PDT efficiency due to the shallow tissue penetration depth. Fortunately, lanthanide‐doped upconversion nanoparticles (UCNPs) can convert low‐energy near‐infrared (NIR) light to high‐energy ultraviolet/visible light, providing a new way to overcome this limitation.^[^ ^115^, ^116^, ^117^ ^]^ The modification of UCNP cores can broaden the application scope of nMOFs. For example, the doping of Eu^3+^ can construct an integrated nanoplatform for NIR‐II imaging, PDT, and immunotherapy.^[^ ^107^ ^]^ Zhao et al. developed a new in situ growth strategy of nMOFs for synergistic PDT and immunotherapy of tumors (Figure 2c).^[^ ^106^ ^]^ By integrating a porphyrin Zr‐based metal–organic framework with lanthanide NaYF4:Gd, Tb@NaYF4 scintillator nanoparticles (SNPs), the obtained nanoprobe can be activated by a soft X‐ray. This soft‐X‐ray‐triggered PDT can not only significantly enhance ROS generation at a tissue depth of 3 cm, but also trigger ICD, eventually activating antitumor immune responses. Recently, Li et al. developed a spindle‐shaped PCN‐222—SO_3_H (PCN‐SU) with enhanced NIR absorption in order to improve the therapeutic effect of NIR irradiation.^[^ ^114^ ^]^ The sulfonating reaction produces strong intramolecular hydrogen bonds between the introduced sulfonic anions and tris(chlorisopropyl)phosphate (TCPP) ligands, inducing the distortion of porphyrin rings, consequently leading to the lower energy gap of highest occupied molecular orbital (HOMO) and lower unoccupied molecular orbital (LUMO), thereby increasing the absorption in the near‐infrared region.

#### 4.3.3 nMOFs for Enhancement of PDT‐*Induced ICD*

Although PDT‐induced ICD can induce host antitumor responses, such responses are generally weak and not enough to eliminate the residue cancer cells. Owing to the porous inner structure of photosensitizer‐formed MOFs, other small molecular drugs or adjuvants like CpG oligodeoxynucleotides can load with high efficiency for combining PDT with other therapeutic modalities to trigger the release of a large number of antigens and induce a strong immune response.^[^ ^102^, ^103^, ^105^, ^110^, ^112^ ^]^ For example, through antigen‐presenting DCs, more CD8^+^ and CD4^+^ T cells are generated, leading to stronger immunotherapy. Ni et al. designed W–TBP, which mediates PDT to release TAAs and delivers immunostimulatory CpG oligodeoxynucleotides to DCs.^[^ ^102^ ^]^ The cationic nanoscale metal–organic framework allows higher loading efficiency of CpG to enhance antigen presentation and expand and reinvigorates cytotoxic T cells in a bilateral breast cancer model when it synergizes with checkpoint blocking immunotherapy (CBI). Cai et al. developed MOF‐based nanoparticles self‐assembled from H_2_TCPP and zirconium ions with hypoxia‐inducible factor (HIF) signaling inhibitor (ACF) and immunologic adjuvant (CpG) loading, and hyaluronic acid (HA) coating on the surface.^[^ ^103^ ^]^ Nanoparticles with CD44 receptor targeting can inhibit HIF‐*α*‐induced tumor survival and metastasis by blocking the hypoxic survival signal aggravated after PDT via ACF.

#### 4.3.4 nMOFs for the Combinational Immunotherapy with PDT and Chemotherapy/CDT

Typically, Shao et al. designed an upconverted metal–organic framework, which contains a heterostructure that allows efficient energy transfer from the UCNP core to the MOF shell and then enables the NIR‐light‐triggered production of cytotoxic ROS.^[^ ^104^ ^]^ The combination of PDT, chemotherapy, and immunotherapy was successfully achieved by loading the hypoxia‐activated prodrug tirapazamine (TPZ) and integrating it with anti‐PD‐L1 therapy. With the assistance of CpG, enhanced PDT can elicit a stronger antitumor immune response. Dendri‐*graft*‐l‐lysine (DGL)‐loaded Gem can also be delivered through nMOFs to deplete MDSCs and reverse the immunosuppressive TME, ultimately enhancing the immune response.^[^ ^105^ ^]^ The ROS generated by PDT is not only able to kill tumor cells but also break the disulfide bond between MOF and macromolecules, thereby realizing targeted deeper delivery of Gem with the help of DGL.

In addition to the ligands acting as photosensitizers in SBU, its metal ions can also integrate other biological functions, such as triggering the Fenton reaction.^[^ ^108^, ^109^, ^111^ ^]^ In this case, further combined immunotherapy can activate a more powerful antitumor effect. Zhao et al. designed nMOFs that consist of vorinostat (SAHA)‐loaded manganese–porphyrin metal–organic framework (Mn(III)–TCPP MOF) with further modification of AS1411 aptamer (Figure 2d–f).^[^ ^109^ ^]^ Mn(III) is reduced to Mn^2+^ by excess glutathione (GSH) in tumor cells, while the nanoparticles were degraded and free TCPP molecules were released. The released Mn^2+^ further enhanced the STING‐pathway‐mediated innate immunity, which synergized with PDT‐induced ICD to achieve coactivation of innate and adaptive immunity. Notably, since TCPP can undergo intracellular self‐assembly with AS1411 and further target the nucleus, PDT mainly occurs in the nucleus and mediates severe nuclear DNA damage and cytosolic release under laser irradiation with the help of SAHA to decompress chromatin, which activated the cytosolic DNA/cyclic GMP–adenosine monophosphate (AMP) synthase (cGAS) pathway at the same time. Ni et al. developed Cu–TBP to combine PDT with CDT.^[^ ^108^ ^]^ After pH‐responsive degradation of the nMOFs in TME, the released Cu^2+^ will catalyze Fenton‐like reactions as well as estradiol‐dependent ROS generation. Therefore, the enhanced radical therapy could synergize with CBI effectively to distant tumors via abscopal effects on mouse tumor models with high estradiol levels.

### 4.4 nMOFs for Combinational Immunotherapy with CDT

Strategies targeting the TME are attracting more and more attention. One of the strategies that have been developed is to exploit the reactivity of ROS and highly expressed GSH in the TME to drive the selective delivery and release of various therapeutic drug molecules to the hyperactivated complex redox environment.^[^ ^118^ ^]^ CDT is based on Fenton or Fenton‐like reactions to convert H_2_O_2_ into hydroxyl radicals triggering apoptosis and inhibiting tumor growth. As an emerging therapy approach, immunotherapy has various limitations. For example, many immunotherapies are ineffective against poorly immunogenic tumors. The combination of CDT and immunotherapy is one of the ways to improve immunotherapy. CDT leads to ROS accumulation and oxidative stress in tumor cells, causing mitochondrial damage and inducing ICD, reversing the immunosuppressive microenvironment.

MOFs are ideal platforms for CDT due to their easy targeting, large capacity, and stable structure.^[^ ^46^ ^]^ Whether synthetic enzymes or natural enzymes carried in MOFs can generate ROS by catalyzing the reaction of oxygen and H_2_O_2_ to achieve effective tumor therapy, simultaneously improving the tumor immunogenicity efficiently.

**Figure 3:**
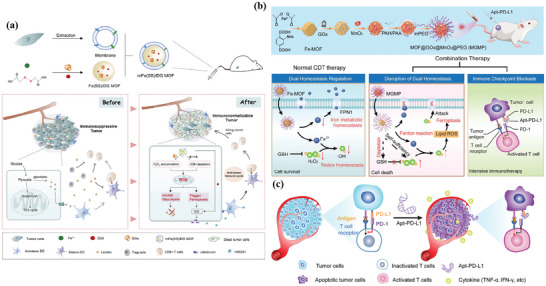
a) Schematic illustration of the smart biomimetic nanoplatform for antitumor chemo‐immunotherapy and immunometabolism normalization based on the ROS–ferroptosis–glycolysis regulation. Reproduced with permission.^[^ ^119^ ^]^ Copyright 2021, Elsevier. b) Schematic synthesis of MOF@GOx@MnO_2_@PEG (MGMP) for ferroptosis‐based immunotherapy. MGMP disrupts redox and iron homeostasis to improve ferroptosis‐based immunotherapy through GSH depletion, ROS production, downregulation of transferrin 1, and PD‐L1 checkpoint block suppression. c) Schematic illustration of Apt‐PD‐L1 blocking immune checkpoint. Reproduced with permission.^[^ ^120^ ^]^ Copyright 2022, Elsevier.

Extensive literature suggests that the metastatic potential of tumor cells is strongly influenced by microenvironmental cues such as hypoxia. Hypoxia is a typical feature of the tumor microenvironment, and it drives the aggressiveness of many tumors. In the process of adapting to the hypoxic tumor microenvironment, cancer cells acquire invasive and metastatic properties. An iron‐based nMOFs loaded with DOX and glucose oxidase (GOx) were designed based on the “ROS–ferroptosis–glycolytic regulation” strategy (**Figure** **3**a).^[^ ^119^ ^]^ Fe^3+^ removes excess GSH in tumor cells and downregulates glutathione peroxide 4 (GPX4) to induce ferroptosis, which can combine with DOX to induce ICD in tumor cells and release TAAs to initiate antitumor immunity. GOx catalyzes the degradation of glucose and generates abundant H_2_O_2_, which enhances the Fe^2+^‐mediated Fenton reaction. As expected, the cancer membrane coated MOF with loading DOX and GOx (mFe(SS)/DG) treatment exhibited the most potent antitumor effect owing to the homologous targeting and the synergy of ROS–ferroptosis–glycolysis regulation and chemotherapy. Furthermore, the explosive production of ROS promotes ferroptosis and glycolysis repression which can remodel tumor immunosuppressive microenvironment (TIME) by decreasing lactate to solidify and boost the antitumor immunity. mFe(SS)/DG elevates the amount of helper CD4^+^ and cytotoxic CD8^+^ T cells compared with other groups, demonstrating the enhancement of antitumor immune responses. The level of CD3^+^/CD8^+^/CD44^+^ T cells in the mFe(SS)/DG group was higher than other groups, indicating the activation of CD8^+^ T cells induced by mFe(SS)/DG. However, here, ferroptosis can effectively induce ICD but is limited by the effect of the Fenton reaction. Zhang et al. established an improved ferroptosis therapy.^[^ ^120^ ^]^ By loading MnO_2_ on MIL‐101(Fe), GSH was depleted and GPX4 expression was downregulated (Figure 3b,c). On the other hand, surface‐loaded GOx generates H_2_O_2_ in situ, and the upregulated H_2_O_2_ will be converted into ROS for downregulating the expression of ferroportin 1, which can transfer unused iron out of cells. Through the dual disruption of redox homeostasis and iron metabolism homeostasis within the TME, cells undergo more efficient ferroptosis and further release tumor antigens. When combined with anti‐PD‐L1, it showed a more powerful immunotherapeutic effect.

Another typical feature of the immunosuppressive microenvironment is the long‐term accumulation of adenosine (Ado) in tumor tissues. A sufficient oxygen supply is a prerequisite for diminishing Ado accumulation by relieving hypoxia, preventing the breakdown of adenosine triphosphate into AMP and the hydrolysis of AMP into Ado. Liang et al. developed calcium‐phosphate‐reinforced iron‐based MOFs to reduce Ado accumulation and inhibit Ado‐mediated immunosuppressive response in the tumor microenvironment.^[^ ^121^ ^]^ Degraded CaP under acidic conditions increases the concentration of free phosphate ions in solution, restoring the activity of adenosine kinase in the TME, which catalyzes the activity of Ado phosphorylation and regulates Ado metabolism. The Fe^2+^ generated by degradation triggers the Fenton reaction in tumor cells, relieves tumor hypoxia, and further hinders the generation of Ado. The synergistic effect of CDT and immunotherapy blocks Ado‐mediated immunosuppression and enhances antitumor immune responses, including increasing T lymphocytes and DCs, and reducing regulatory T lymphocytes.

### 4.5 nMOFs for Combinational Immunotherapy with PTT

PTT is a novel antitumor strategy that exhibits efficient thermal ablation of tumor cells. Using the photothermal effect of photothermal transducers, PTT can harvest energy from light and convert the energy into heat, thereby increasing the temperature of the surrounding environment and triggering cancer cell death.^[^ ^122^, ^123^ ^]^ During the thermal ablation, due to the concomitant expression of damage‐associated pattern molecules (DAMPs) (heat shock protein 70, calreticulin, adenosine triphosphate, etc.) and the release of TAAs from tumor cell remnants, the cellular remnants produced by thermal ablation are highly immunogenic and can trigger sufficient immune response activation and induce antigen‐specific immunity against tumors.^[^ ^124^, ^125^ ^]^

Metal–organic frameworks are emerging as one of the most promising light‐responsive materials because their structure and chemical composition can be easily tuned to achieve specific functions. With a rational structural design, MOFs can have potential photothermal capabilities or serve as carriers for photothermal agents and immune adjuvants due to their tunable porosity, providing feasibility for targeted approaches and combined immunotherapy, thereby improving the efficiency of PTT.^[^ ^126^ ^]^ The MOFs for combinational immunotherapy with PTT or CDT or RT/RDT are summarized in **Table** **3**.

**Table 3:** The MOFs for co‐immunotherapy with PTT or CDT or RT/RDT

NPs	Type of MOF	Target	Stimuli‐responsive cargo release	Payload and encapsulation	Applications	Ref.
mFe(SS)/DG	Fe^3+^‐dithiodiglycolic acid	Homotypic targeting (by coating of cancer cell membrane)	ROS‐responsive (ROS generated by GOx breaks disulfide bonds)	Glucose oxidase (GOx) and DOX	Immunotherapy/CT/CDT	[119]
CaP@Fe–MOFs	Fe–TCPP	Tumor cells	pH‐responsive	–	Immunotherapy/CDT	[121]
MOF@GOx@MnO_2_@PEG	MIL‐101(Fe)	Tumor cells	GSH‐responsive	MnO_2_ and GOx	Immunotherapy/CDT	[120]
Dual tailor‐made MOF (HA/IR820@ZIF‐8 and MAN/(R837+1 MT)@ZIF‐8)	ZIF‐8	Tumor cells (by modification of HA)	pH‐responsive	IR820	Immunotherapy/PTT	[127]
		DCs by modification of MAN)		R837 and 1‐methyl‐d‐tryptophan (1 MT)		
ICG–CpG@MOF	MIL‐101—NH_2_	Tumor cells	pH‐responsive	Indocyanine green (ICG) and CpG	Immunotherapy/PTT/PDT	[128]
CuCo(O)/GOx@PCNs	Cu/ZIF‐8@ZIF‐67	Tumor cells	pH‐responsive	Co_3_O_4_, Gox, N‐doped carbon nanotube hollow sphere	Immunotherapy/PTT/ starvation	[129]
MIL‐100 loaded with MTO and HA as nanoparticles (MMH NPs)	MIL‐100	Tumor cells (by modification of HA)	pH‐responsive	Mitoxantrone (MTO)	Immunotherapy/PTT/CT	[130]
MPSNs@R837	Zn^2+^‐porphyrin	Tumor cells	pH‐responsive	Imiquimod (R837)	Immunotherapy/PTT/PDT	[131]
ICG@ZIF‐8(Al) NPs	ZIF‐8	Tumor cells	pH‐responsive	ICG, Al^3+^	Immunotherapy/PTT	[132]
MIL‐100 NPs with oxaliplatin (OXA) and indocyanine green (ICG) loading and hyaluronic acid (HA) modification (OIMH) NPs	MIL‐100 (Fe)	Tumor cells (by modification of HA)	pH‐responsive	Oxaliplatin (OXA) and ICG	Immunotherapy/PTT/CT	[133]
Hf–DBP/TBP	Hf–DBP/TBP	Tumor cells	–	iIDO	Immunotherapy/RT/RDT	[134]
Hf_6_DBA/Hf_12_DBA	Hf_6_DBA/Hf_12_DBA	Tumor cells	–	–	Immunotherapy/RT/RDT	[135]
Hf–DBP–Fe	Hf–DBP (doped by Fe^3+^)	Tumor cells	–	–	Immunotherapy/RT/RDT/CDT	[136]
Hf–DBB^F^–Ir	Hf–DBB–Ir	Tumor cells	–	CpG	Immunotherapy/RT/RDT	[137]

**Figure 4:**
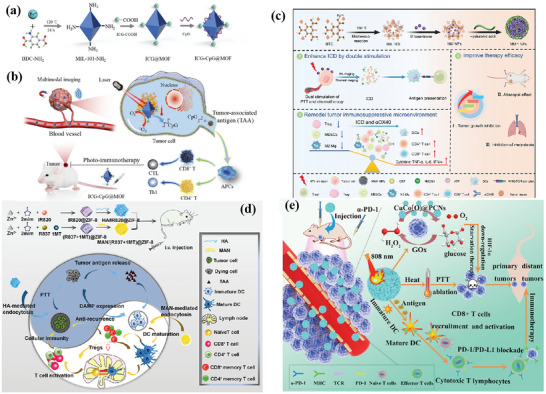
Schematic illustration for a) synthesis of ICG–CpG@MOF and b) mechanism of multimodality‐imaging (photoacoustic, nuclear magnetic, fluorescence imaging)‐guided synergistic cancer photo‐immunotherapy. Reproduced with permission.^[^ ^128^ ^]^ Copyright 2020, Elsevier. Schematic illustration of c) the routine characterization and the photo‐chemo‐immunotherapy strategy of the MMH NPs. Reproduced with permission.^[^ ^130^ ^]^ Copyright 2021, American Chemical Society. d) Illustration of the synthesis of HA/IR820@ZIF‐8 and the MAN/(R837 + 1 MT)@ZIF‐8 nanoparticles and the mechanisms of the combination effects of PTT and antitumor immunotherapy induced by these mixed nanoparticles. Reproduced with permission.^[^ ^127^ ^]^ Copyright 2020, Elsevier. e) Schematic Illustration of the ZIF‐derived nanozyme CuCo(O)@PCNs with three‐in‐one functions to achieve synergetic therapy. Reproduced with permission.^[^ ^129^ ^]^ Copyright 2021, American Chemical Society.

Fan et al. constructed a multimodal‐imaging‐guided synergistic cancer photoimmunotherapy with MIL‐101—NH_2_ as the core carrier (**Figure** **4**a,b).^[^ ^128^ ^]^ Indocyanine green (ICG) loading integrates photoimmunotherapy and multimodal imaging of tumors (fluorescence, photoacoustic, photothermal, and magnetic resonance imaging). The temperature of ICG–CpG@MOF increased sharply and reached equilibrium within 5 min to 82 °C after irradiation with an 808 nm laser. After tail vein injection for 6 h, the NPs caused strong photoacoustic signals in vivo. Under the irradiation of 808 nm laser, PDT and PTT were simultaneously activated to induce ICD and release TAAs. With the assistance of CpG released by MOF, PTT successfully activated the immune system and enhanced the antitumor effect. However, these nanoparticles only passively accumulate at the tumor site through the EPR effect, and the lack of targeting will affect the PTT and imaging results. To improve the targeting, Ni et al. surface‐modified MIL‐100 with HA for targeted delivery of nMOFs. At the same time, the released chemotherapeutic agent mitoxantrone (MTO) can bind to PTT and enhance ICD (Figure 4c).^[^ ^130^ ^]^ This dual therapy and dual imaging (photoacoustic and thermal) properties resulted in the highest antitumor efficacy and potent immune effects when MMH NPs were coinjected with anti‐OX40 antibodies in colorectal cancer. The MMH NPs + laser group temperature reached much higher (56.6 ± 0.15 °C) than the phosphate‐buffered saline (PBS) + laser group (42.9 ± 0.65 °C), which indicates that MMH NPs have good photothermal properties in vivo. The work of Liu et al. used a similar principle, except that oxaliplatin (OXA) was chosen as the therapeutic agent.^[^ ^133^ ^]^ Another approach to improve targeting is to use MOFs to achieve specific release of drug molecules. Li et al. applied one‐pot synthesis to introduce a second metal to produce bimetallic MOFs. The synthesized ZIF‐8(Al) can be used to encapsulate ICG, and the aluminum ion itself can be used as an immune adjuvant to amplify the tumor‐specific immune response.^[^ ^132^ ^]^ Under laser irradiation, nanoparticles can effectively induce tumor cells to develop ICD and release TAAs. Activation of antitumor immunity with the assistance of aluminum adjuvant and the generation of long‐term immune memory shows an efficient antirecurrence effectin‐treated mice after a tumor cell rechallenge.

Antitumor immune response was a complex process involving many types of immune cells. Therefore, synergistically modulating the functions of multiple immune cells involved in the immune process based on the spatiotemporal cooperation of various therapeutic agents can be an efficient strategy. Zhang et al. designed a customized drug delivery system (ZIF‐8) with specific modifications to enable active targeted delivery of each drug to different on‐demand sites (Figure 4d).^[^ ^127^ ^]^ The IR820‐loaded ZIF‐8 nanoparticles were modified with HA to accomplish the active targeting delivery of HA/IR820@ZIF‐8 toward tumor cells for improving the expression of DAMPs and TAAs to trigger an immune response. By contrast, the imiquimod (R837)‐ and 1‐methyl‐d‐tryptophan (1 MT)‐coloaded ZIF‐8 nanoparticles were modified with mannan (MAN) (MAN/(R837 + 1 MT)@ ZIF‐8) whose receptors were highly expressed on the surface of DCs, realizing DC‐targeted drug release to enhance the antitumor immunity further. The photothermal imaging pictures showed that the MMH NPs + laser group temperature reached much higher (56.6 ± 0.15 °C) than the PBS + laser group (42.9 ± 0.65 °C), which indicates that MMH NPs have good photothermal properties and thermal imaging capability in vivo. Tumor autoantigens generated after photothermal ablation could show vaccine‐like functions in combination with immune adjuvants to effectively motivate systemic antitumor immunity, and the immunomodulation mediated by 1 MT on immunosuppressive IDO could significantly increase the ratio of effector T cells (CD8^+^/CD4^+^ T cells) to immunosuppressive Tregs, thereby preventing immune evasion, and consequently promoting the secretion of proinflammatory factors Interferon *γ* (IFN‐*γ*) and IL‐6, and effectively activate systemic immunity.

Carbon complexes have been made to be NIR‐light‐responsive PTT agents owing to the high photothermal transformation efficiency in a NIR biological window. Wang and co‐workers developed a three‐in‐one ZIF‐derived CuCo(O)/GOx@PCN hybrid cascade nanozyme in view of the photothermal properties of carbon nanomaterials and the catalase‐like properties of transition metal oxides such as Co_3_O_4_ (Figure 4e).^[^ ^129^ ^]^ A synergistic effect of PTT and enhanced starvation therapy can be expected if GOx is loaded into carbon nanomaterials. Cancer‐related antigens caused by the synergetic therapy activated the immune response and after that the nanozyme played the *α*‐PD‐1‐antibody‐like role and activated the inhibited T cells, resulting in the distant tumor cells killed by cytotoxic T cells.

### 4.6 nMOFs for Combinational Immunotherapy with RT and RDT

RT and RDT destroy tumor tissue using ionizing radiation and generate damaging hydroxyl radicals.^[^ ^32^ ^]^ RT induces ICD, and the released DAMPs promote the phagocytosis of tumor antigens by DCs, and ultimately stimulate the maturation and differentiation of DCs. This process allows tumor antigens to be presented to T lymphocytes through the formation of MHC‐I complexes, promoting the proliferation of toxic T lymphocytes and infiltration of tumor sites.^[^ ^138^ ^]^ RT promotes the delivery of TAAs and tumor DNA to DCs, which activates type I IFN production via the STING pathway.^[^ ^139^ ^]^ Thus, RT stimulates a potential systemic immune response under the combined effects of innate and adaptive immunity.

Since lower doses of radiation often fail to elicit a strong enough immune response, but higher doses can damage off‐target tissue, Lu et al. have explored the use of nMOFs as radioenhancers in combination with low‐dose radiation. The hafnium (Hf)‐based nMOF was synthesized through coordination between Hf_12_O_8_(OH)_14_ and 2,5‐di(*p*‐benzoato)aniline.^[^ ^134^ ^]^ The efficient absorption of X‐ray photons by Hf clusters leads to RT (by generating OH radicals) and RDT (by exciting photosensitizers to generate ^1^O_2_). Consequently, the original vaccination was achieved. When used in combination with immune checkpoint inhibitor molecules, primary tumors were eradicated and distal tumors were rejected through abscopal responses. Similarly, this group designed two Hf‐based nMOFs, Hf_6_–DBA and Hf_12_–DBA (DBA = 2,5‐di(*p*‐benzoato)aniline) as radiosensitizers.^[^ ^135^ ^]^ As a result, Hf_12_–DBA has a stronger antitumor efficacy due to increased X‐ray absorption and ROS diffusion more likely due to the thin nanoplate structure leading to increased ROS production. Combination with anti‐PD‐L1 antibody can not only eradicate local tumors, thanks to abscopal effects, but also reject/regress distant tumors by systemic antitumor immunity.

**Figure 5:**
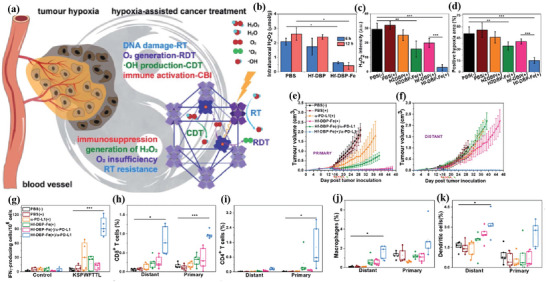
a) Scheme, Hf–DBP–Fe harnesses tumor hypoxia for cancer treatment via RT–RDT and CDT as well as synergistic combination with CBI. Local injection of Hf–DBP–Fe in MC38 tumors followed by low‐dose X‐ray irradiation relieves tumor hypoxia by porphyrin–Fe(III)‐mediated H_2_O_2_ decomposition to generate O_2_ for RDT and cOH for both CDT and RT. ICD caused by local RT–RDT and CDT synergizes with anti‐PD‐L1 CBI to lead to systemic antitumor immunity. b) H_2_O_2_ concentration of tumors treated with PBS, Hf–DBP, or Hf–DBP–Fe at an equivalent dose of 0.2 mmol per mouse 6 or 12 h after injection quantified with TiOSO_4_, *n* = 3. Quantitative analysis of c) the total fluorescence intensity of the H_2_O_2_ kit, d) the positive area of hypoxia. e) Primary and f) distant tumor growth curves of MC38 bilateral tumor‐bearing mice treated with different groups. g) Enzyme‐linked Immunospot (ELISPOT) assay for the detection of tumor‐specific IFN‐g producing T‐cells. Splenocytes were harvested 12 days after the first treatment and were cocultured with the stimulating KSPWFTTL peptide for 42 h. Percentages of tumor‐infiltrating h) CD8^+^ T cells, i) CD4^+^ T cells, j) macrophages, and k) DCs with respect to the total tumor cells, calculated by flow cytometric analysis. Reproduced with permission.^[^ ^136^ ^]^ Copyright 2020, The Royal Society of Chemistry.

From the above results, it can be seen that the effect of RT will be affected by tumor hypoxia. Therefore, combining other therapies to alleviate intratumoral hypoxia will improve the treatment efficiency of RT/RDT. Ni et al. developed Hf–DBP–Fe, with catalase‐like activity, to decompose elevated levels of H_2_O_2_ in hypoxic tumors to generate oxygen and hydroxyl radicals (**Figure** **5**a–d).^[^ ^136^ ^]^ The generated oxygen attenuates hypoxia to enable RDT upon X‐ray irradiation and fixes DNA damage while hydroxyl radical inflicts direct damage to tumor cells to afford CDT. The synergistic combination of Hf–DBP–Fe‐mediated CDT and RT–RDT with anti‐PD‐L1 resulted in excellent antitumor results in a bilateral syngeneic model of colon cancer (Figure 5e,f), expanding the local efficacy of CDT and RT–RDT in untreated tumors by systemic anticancer immunity (Figure 5g–k).

Combination with immune adjuvants can activate a more robust immune response and prevent advanced tumors from evading immune surveillance. Ni et al. reported a novel Hf‐based cationic nMOF, Hf–DBB^F^–Ir (DBB = 4,4′‐di(4‐benzoato)‐2,2′‐ bipyridine), by mediating efficient RT–RDT to generate immunogenic tumor antigens and DAMPs and deliver anions in pathogen‐associated molecular patterns (PAMPs) CpG for nonviral in situ vaccination.^[^ ^137^ ^]^ The results demonstrate that DAMP‐induced cGAS–STING and CpG‐induced TLR pathways such as PAMP operate independently, suggesting that they may be activated simultaneously to achieve additive or synergistic effects on immune stimulation. It has been proven that the nMOF‐based cancer vaccine extends the local therapeutic effect of an in situ cancer vaccine to distant tumors by reactivating systemic antitumor immunity of CTLs in a bilateral MC38 tumor model.

## 5 MPNs for Cancer Immunotherapy

MPNs are supramolecular network structures established by the coordination interaction between metal ions (e.g., Fe^3+^, Mn^2+^, and Gd^3+^) and polyphenols (e.g., natural polyphenols and synthetic polyphenols), which combine specific functions of metal ions with the high affinity of phenolics to a wide range of surfaces.^[^ ^32^ ^]^ Polyphenols existing widely in natural plants have been demonstrated to have the effect of antitumor, antioxidation, antiradiation, and antithrombosis, and most of which have been FDA‐approved for food preparation and human health.^[^ ^33^ ^]^ Most importantly, polyphenols remain abundant in phenolic moieties that can chelate metal ions, while metal ions play a vital role in biomedicine and chemical catalysis. The plentiful types of metal ions and polyphenols endow MPNs with diverse properties and functions. Additionally, the pH‐responsive character of MPNs allows for disassembly kinetics to facilitate the release of metals and polyphenols. Therefore, as an emerging new class of coordination materials, MPNs have been explored as multifunctional nanoplatforms for various biomedical applications due to specific functional moieties, redox‐responsive behavior, simple synthesis, and benign affinity with body tissues.^[^ ^32^ ^]^ In this section, we focus on the important role of MPNs in cancer immunotherapy including vaccine delivery and MPN‐mediated RT, RDT, PTT, and SDT. **Table** **4** summarized the MPNs used for cancer immunotherapy.

**Table 4:** Summary of the research on MPNs for cancer immunotherapy

Type	Diameter	Metal	Phenolic ligands	Payloads	Administration route	Tumor model	Ref.
Vaccine	90 nm	Fe^3+^	Catechol‐terminated eight‐arm PEG (CPEG)	PMSN@OVA	s.c.	E.G7‐OVA	[140]
CT	100–200 nm	Fe^3+^	TA	Carfilzomib, albumin	i.t.	B16F10, CT26	[141]
CT	80 nm	Pt^2+^, Fe^3+^	TA	–	i.v.	CT26	[142]
CDT	60–100 nm	Fe^3+^	Epigallocatechin‐3‐gallate (EGCG), 5‐hydroxydopamine modified platinum(IV) prodrug (Pt—OH), PEG‐*b*‐PPOH	–	i.v.	HepG2	[143]
CDT	28 nm	Fe^3+^	PEG–polyphenols	DOX, MnO_2_	i.v.	B16F10	[144]
CDT	45 nm	Fe^3+^	TA	DOX, fibronectin	i.v.	B16	[145]
RT/RDT	30 nm	Hf^4+^	Ce6–PEG–polyphenols	Hemoglobin	i.v.	4T1	[146]
RT	21 nm	Hf^4+^	Ce6–PEG–polyphenols	Atovaquone, sabutoclax	i.v.	4T1	[147]
RT	82 nm	Hf^4+^	Polyphenolic semiconductor polymer	PEG–DTC, PLX	i.v.	4T1	[148]
RT	7 nm	Mn^2+^, NaGdF_4_:Nd@NaLuF_4_	PEG–polyphenols	–	i.v.	4T1	[149]
PTT	194 nm	Fe^3+^	Gallic acid (GA)	BSA, OVA, or HSA	i.v.	4T1	[150]
PTT	80 nm	VO^2+^	TA	Silk sericin	i.t.	B16F10	[151]
PTT	149–168 nm	Mn^2+^, Fe^3+^	TA	PD‐L1 inhibiting DNAzyme	i.v.	B16F10	[152]
PTT	80 nm	Fe^3+^	Phenolic semiconductor polymer, PEG–semiconductor polymer	GW4869	i.v.	B16F10	[153]
PTT	100–200 nm	Fe^3+^	pTA	PLGA	i.t.	4T1	[154]
PDT	32 nm	Fe^2+^	Gossypol, PEG–Ce6	–	i.v.	4T1	[155]
SDT	68 nm	Mn^2+^	PEG‐*b*‐Pho, PEG‐*b*‐IR	Sabutoclax	i.v.	4T1	[156]
IRE	40 nm	Mn^2+^	TA	CpG–ODN	i.t.	CT26	[157]

### 5.1 MPNs for Vaccine Delivery

**Figure 6:**
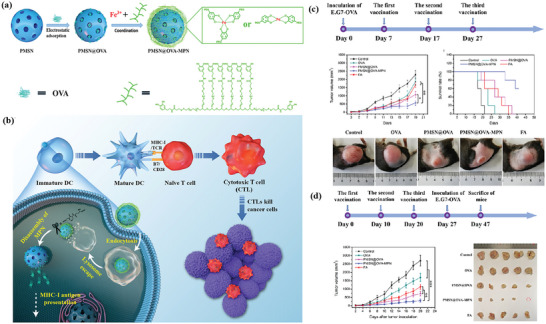
a) Schematic illustration of preparation of PMSN@OVA–MPN. b) Process of PMSN@OVA–MPN in vivo eliciting cellular immune responses via the MHC‐I pathway and generated CTL killing tumor cells. c) In vivo antitumor immunotherapeutic results. d) Evaluation of prophylaxis efficacy against an E.G7‐OVA tumor. Reproduced with permission.^[^ ^140^ ^]^ Copyright 2020, American Chemical Society.

Cancer nanovaccines have been widely explored to enhance immunotherapy efficiency and the effect of MPNs as a protective layer for nanovaccine delivery systems was also explored. The strong metal polyphenol coordination interactions lead to the high stability of MPNs during circulation stems. In 2020, Zhou et al. developed a pH and reduction dual‐sensitive nanovaccine composed of polyethyleneimine (PEI)‐modified MSNs (PMSN) loaded with model antigen OVA and the outer part was disulfide‐bond‐involved MPNs as a protective corona (PMSN@OVA‐MPN) (**Figure** **6**a).^[^ ^140^ ^]^ When subcutaneously injected into the body, MPNs were disintegrated by glutathione, and OVA was released into the cytosol and presented by the MHC‐I pathway to induce cellular immune response and generation of CTLs (Figure 6b). In vivo animal experiments indicated that PMSN@OVA–MPN induced a large tumor‐specific cellular immune response to effectively inhibit tumor growth (Figure 6c,d). Hence, the MPN‐mediated multifunctional vaccine delivery system exhibits a great application potential in cancer immunotherapy and offers a platform for the development of nanovaccines.

### 5.2 MPNs for Combinational Immunotherapy with Chemotherapy and CDT

**Figure 7:**
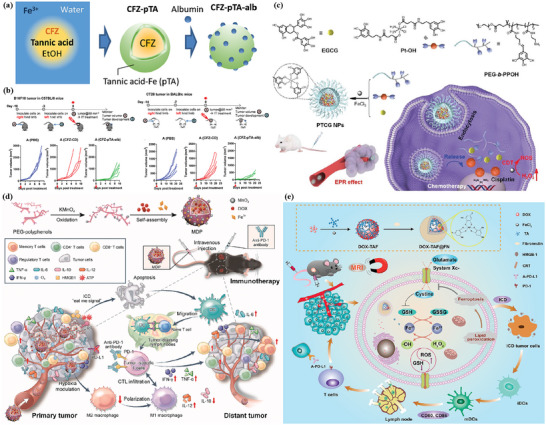
a) Schematic description of CFZ–pTA preparation by TA/Fe^3+^ interfacial assembly formation and CFZ–pTA–alb with albumin coating. b) Effect of CFZ–pTA–alb versus CFZ–CD, administered as a single intratumoral injection at a dose equivalent to 60 µg CFZ, on B16F10@C57BL/6 mice and CT26@Balb/c mice. Reproduced with permission.^[^ ^141^ ^]^ Copyright 2019, American Chemical Society. c) The chemical structures and cartoon illustration of the building blocks (EGCG, Pt—OH, PEG‐*b*‐PPOH) used for the preparation of PTCG NPs, and high tumor accumulation of PTCG NPs through EPR effect followed by the cellular internalization. Reproduced with permission.^[^ ^143^ ^]^ Copyright 2019, Wiley‐VCH. d) Schematic illustration of a phenolic immunogenic cell death inducer for sensitizing tumor to PD‐1 checkpoint blockade immunotherapy and its immune mechanisms. Reproduced with permission.^[^ ^144^ ^]^ Copyright 2020, Elsevier. e) Formation of DOX–TAF@FN nanocomplexes for in vivo MRI and combined chemo‐/chemodynamic/immune therapy of tumors. Reproduced with permission.^[^ ^145^ ^]^ Copyright 2022, American Chemical Society.

To achieve the targeted delivery of drugs to tumors, recent studies have shown that the MPNs could be used to load chemotherapeutic drugs in their hydrophobic cavities and extend blood circulation time. As a natural phenolic ligand, tannic acid (TA) is already approved by the USFDA. TA exhibits antioxidant, antibacterial,^[^ ^158^ ^]^ and anticarcinogenic^[^ ^159^ ^]^ properties and can chelate various metal ions. For example, Taha et al. developed a supramolecular (CFZ–polytannic acid (pTA)–alb) assembly of TA and Fe^3+^ to encapsulate ICD‐inducing proteasome inhibitor carfilzomib (CFZ), and modified the surface with albumin to control the drug release. The adhesive nature of the TA assembly could capture tumor antigens and DAMPs to enhance their delivery to DCs and prolong tumor retention of CFZ in B16F10 and CT26 tumor models (**Figure** **7**a,b).^[^ ^141^ ^]^ Compared to cyclodextrin‐solubilized CFZ (CFZ–CD), CFZ–pTA–alb enhanced the population of CD8^+^ T cell in tumors and develop splenocytes with tumor‐specific IFN‐*γ* response. Traditional cancer chemotherapy has limitations, including low bioavailability, high lethality to normal tissues, and rapid clearance. CDT has been considered as an efficient strategy for cancer treatment to destroy cancer cells by converting H_2_O_2_ into highly toxic reactive oxygen species, which is also called Fenton chemistry. Natural polyphenols can be used as carriers for pharmaceutically active metals through complexation. A facile preparation was proposed to use natural polyphenols to build platinum nanocomplex with stable structure, proper size, and high Pt content to trigger ICD.^[^ ^142^ ^]^ Fe(III) was also introduced into the polyphenols–Pt(II) framework to adjust the stability of nanoparticles via multiple Fe(III)–polyphenol complexations. The metal–organic coordination interactions may endow the drug delivery system with an acidity‐ and ROS‐sensitive dissociation and drug release. Some chemotherapeutic drugs like cisplatin have been verified to have the ability to induce CDT.^[^ ^160^ ^]^ Ren et al. fabricated the MPNs by incorporating epigallocatechin‐3‐gallate (EGCG), 5‐hydroxydopamine‐modified platinum(IV) prodrug (Pt—OH), and polyphenol‐modified block copolymer (polyethylene glycol (PEG)‐*b*‐PPOH) on the basis of the coordination interactions between Fe^3+^ and polyphenols. The activated cisplatin elevates the intracellular H_2_O_2_ level and further produces ROS catalyzed by iron‐based Fenton reaction. More importantly, the toxicity of cisplatin is avoided by taking advantage of these complex nanoparticles in vivo investigations (Figure 7c).^[^ ^143^ ^]^ In 2021, Xie et al. designed MPNs constructed via metal phenolic coordination of phenolic MnO_2_, Fe^3+^, and PEG–polyphenols, further encapsulating with DOX to induce the ICD effect (Figure 7d).^[^ ^144^ ^]^ In 2022, Xu et al. also presented a DOX–TAF@FN nanoparticles through the coordination of TA and Fe^3+^ and physically coated with fibronectin. Fe^3+^ can be used for CDT to induce ferroptosis of cancer cells and synergy with DOX chemotherapy and ultimately enhance tumor‐specific T‐cell infiltration (Figure 7e).^[^ ^145^ ^]^ In comparison with the vast majority of traditional drug delivery systems, the MPNs greatly improve the anticancer efficacy and enrich the functions of the resultant nanomedicine for their character and stability.

### 5.3 MPNs for Combinational Immunotherapy with RT and RDT

**Figure 8:**
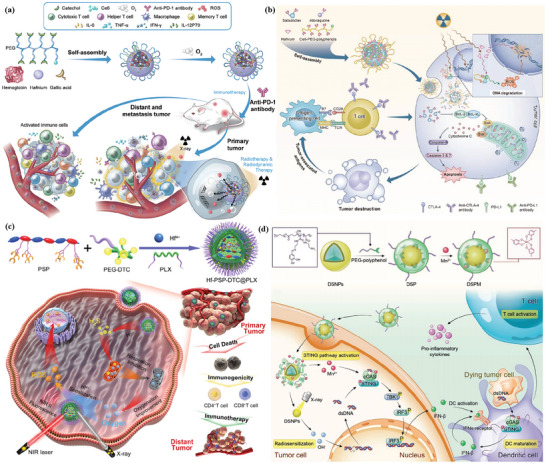
a) Schematic illustration of Hb@Hf–Ce6‐nanoparticle‐mediated X‐ray induced RT–RDT–immunotherapy for the eradication of primary and distant tumors. Reproduced with permission.^[^ ^146^ ^]^ Copyright 2020, Wiley‐VCH. b) Preparation of AHSC NPs and the triple therapeutic strategy for tumor eradication. Reproduced with permission.^[^ ^147^ ^]^ Copyright 2022, Wiley‐VCH. c) Schematic illustration of the NIR‐II fluorescence traced Hf–PSP–DTC@PLX nanosensitizer that performs H_2_S‐reprogrammed oxygen metabolism for RT intensification and immunogenicity. Reproduced with permission.^[^ ^148^ ^]^ Copyright 2022, Wiley‐VCH. d) Schematic illustration of the preparation and biological function of NaGdF_4_:Nd@NaLuF_4_@PEG‐polyphenol/Mn (DSPM). Reproduced with permission.^[^ ^149^ ^]^ Copyright 2022, Wiley‐VCH.

RT based on DNA damage and ROS generation occupies a crucial approach in various types of cancer. Especially, the immunomodulatory adjuvant feature of RT has been identified to boost immunotherapeutic performance.^[^ ^161^ ^]^ However, the application of RT is greatly limited by toxicity, nonselectivity, and hypoxia‐associated radioresistance. One promising approach is to deliver oxygen directly with hemoglobin (Hb), an oxygen‐transport metalloprotein in the red blood cells.^[^ ^146^ ^]^ MPNs can encapsulate Hb as a nanoparticle framework to reduce the toxicity and immunogenicity of Hb. Hf as a high‐*Z* metal exhibits a superior photoelectric effect upon X‐ray excitation used as a radiosensitizer. In 2021, Sang et al. developed an X‐ray nanoprocessor (Hb@Hf–chlorine e6 (Ce6) NPs) based on metal–phenolic coordination.^[^ ^146^ ^]^ Hb@Hf–Ce6 NPs were fabricated via self‐assembly coordination of Hf and Ce6–PEG–polyphenols with Hb encapsulation for oxygen delivery (**Figure** **8**a). Hb@Hf–Ce6 NPs also activated photosensitizer Ce6 with the assistance of efficient radioluminescence for RT–RDT in combination with immunotherapy. In addition, they also designed a radiosensitizer hafnium coordinated with PEG polyphenols (AHSC NPs).^[^ ^147^ ^]^ The photosensitizer Ce6 conjugated on PEG could be activated by the energy from X‐ray and induced the production of ROS. AHSC NPs embedded with atovaquone and sabutoclax could alleviate hypoxia, accelerate the activation of the apoptosis signaling pathway, and be combined with anti‐CTLA‐4 and anti‐PD‐L1 antibodies to achieve remarkable antitumor capacity (Figure 8b). In 2022, Li et al. designed MPNs that combined an acid‐sensitive hydrogen sulfide (H_2_S) donor (polyethylene glycol‐co‐polydithiocarbamates, PEG–DTC) and a hafnium‐chelated polyphenolic semiconducting polymer (Hf–PSP) in an amphiphilic polymer (poloxamer F127, PLX).^[^ ^148^ ^]^ H_2_S can be released from PEG–DTC in acidic TME to trigger mitochondrial respiration inhibition and inhibit oxygen metabolism for tumor oxygenation improvement. Then, Hf sensitization could utilize the well‐preserved oxygen to intensify RT efficacy and activate immunogenicity (Figure 8c). Yan et al. fabricated the amphiphilic polymer PEG–polyphenol and lanthanide‐doped radiosensitizer‐based MPNs and Mn^2+^ were introduced to activate the STING pathway.^[^ ^149^ ^]^ Upon X‐ray irradiation, MPNs promoted cancer cells’ cytosolic double‐stranded DNA (dsDNA) release and Mn^2+^ facilitated the recognition of cytosolic dsDNA by cGAS, triggering the activation of the STING pathway in both cancer cells and DCs to optimize RT (Figure 8d). To conclude, MPNs enhance cancer immunotherapy through metal‐based or phenolic‐based nanoenhancers to improve the efficacy of RT.

### 5.4 MPNs for Combinational Immunotherapy with PTT

PTT utilizes optical absorbing materials to ablate tumors by converting NIR laser irradiation into hyperthermia, which prompts the extracellular release of DAMPs to trigger the maturation of antigen‐presenting cells such as DCs. The structural and chemical diversity of MPNs make them useful for PTT and possess excellent photothermal efficiency. Gallic acid (GA) is a type of organic tea polyphenol derived from plant sources and has been widely used as an antioxidant additive in foods.^[^ ^162^ ^]^ When GA is coordinated with Fe^3+^, the Fe–GA possesses intense NIR absorption due to the strong delocalization *π*‐electron structure.^[^ ^163^ ^]^ In 2019, Wang et al. designed a bovine serum albumin–Fe–GA nanonetworks with outstanding biocompatibility and excellent photothermal performance for PTT. They also constructed OVA–Fe–GA nanonetworks that successfully induced the maturation of DCs and macrophage cells.^[^ ^150^ ^]^

**Figure 9:**
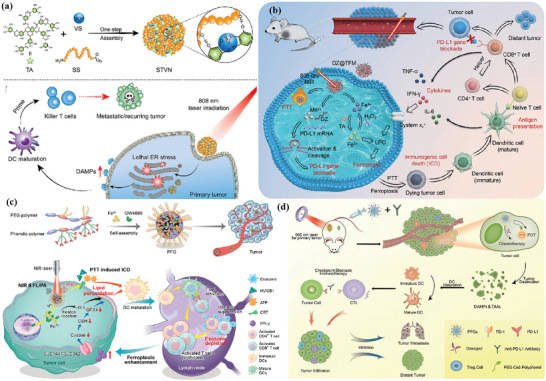
a) Schematic illustration of preparation of vanadyl nanocomplex (STVN), and STVN‐mediated PTT enhances immunogenicity for systemic antitumor immune responses. Reproduced with permission.^[^ ^151^ ^]^ Copyright 2021, Elsevier. b) Schematic illustration of MPNs coordinated with TA and metal‐ion complex of Fe_3+_/Mn^2+^ loaded with a PD‐L1 inhibiting DNAzyme (DZ@TFM) for PTT‐enhanced cyclically amplified tumor ferroptosis–immunotherapy. Reproduced with permission.^[^ ^152^ ^]^ Copyright 2021, Wiley‐VCH. c) Schematic illustration of semiconductor polymers encapsulated Fe^3+^ and exosome inhibitor GW4869 (PFG MPNs) for phototheranostic effect, relief of exosomal immunosuppression, ferroptosis enhancement, and immune stimulation. Reproduced with permission.^[^ ^153^ ^]^ Copyright 2022, American Chemical Society. d) PFGs combined with PD‐L1 checkpoint blockade for immune response enhancement inhibiting tumor proliferation and metastasis. Reproduced with permission.^[^ ^155^ ^]^ Copyright 2020, Wiley‐VCH.

Vanadium‐based photothermal agents (PTAs), including vanadium oxides, have emerged for their substantial light absorption property in the NIR windows.^[^ ^164^ ^]^ In 2021, Hu et al. presented a vanadyl nanocomplex STVN, in which vanadyl ions (VO^2+^) chelated with TA simultaneously integrating a biocompatible protein SS (**Figure** **9**a).^[^ ^151^ ^]^ STVN serves as a PTA and an ICD inducer to enhance the antitumor immunity of PTT and NIR‐irradiated STVN almost entirely ablates primary tumors and inhibits distant tumors in mice bearing bilateral melanoma. In addition, Liu et al. constructed photothermal MPNs that coordinate TA with the metal‐ion complex of Fe^3+^/Mn^2+^, loaded PD‐L1 inhibiting DNAzyme to regulate the immunosuppressive PD‐1/PD‐L1 pathway (Figure 9b).^[^ ^152^ ^]^ Fe^2+^ is generated from Fe^3+^ by TA reduction to trigger ferroptosis and DNAzyme is activated by Mn^2+^ to silence PD‐L1 effectively. PTT is initiated to synergize with ferroptosis for enhanced ICD to induce strong antitumor immunes. Combined with PD‐L1 suppression, PTT obliterates both primary and distant tumors. In 2022, Xie et al. engineered phototheranostic MPNs by an assembly of semiconductor polymers encapsulating ferroptosis inducer (Fe^3+^) and exosome inhibitor (GW4869), which could be used for precise PTT. Excessive secretion of PD‐L1 results in therapeutic resistance to PD‐1/PD‐L1 immunotherapy and clinical failure (Figure 9c).

Therefore, PTT‐augmented ICD effect relieved exosomal silencing on DC maturation, then GW4869‐mediated PD‐L1‐based exosomal inhibition revitalized T cells and enhanced the ferroptosis, which evoked antitumor immunity in B16F10 tumors and immunological memory against metastatic tumors in lymph nodes.^[^ ^153^ ^]^ Recently, pTA‐coated poly(lactide‐co‐glycolide acid) (PLGA) nanoparticles (PLGA–pTA NPs) were synthesized for combined photothermal–immunotherapy. pTA was a coordination complex formed by TA and Fe^3+^, which is a potential photothermal agent since it could convert the NIR light into heat with high efficiency. Hence, PLGA–pTA‐based PTT could effectively trigger DC maturation to enhance therapeutic efficacy by inducing the release of DAMPs.^[^ ^154^ ^]^

### 5.5 MPNs for Combinational Immunotherapy with PDT

As an efficient ICD inducer, PDT can induce quick photodynamic cancer cell death. The most important is that PDT merely concentrates directional and nonionizing radiation on tumor area, which prompts the extracellular release of DAMPs. Natural gossypol stems from the cotton plant and has strong binding affinity to apoptosis‐associated proteins.^[^ ^165^ ^]^ Zhang et al. used natural polyphenol gossypol and PEG–Ce6 to coordinate to Fe^2+^, forming a nanoplatform for the combination of PDT (Figure 9d). The combination of gossypol and the photosensitizer Ce6 can induce chemotherapeutic and photodynamic ICD effects upon laser irradiation to induce the maturation of DCs and the secretion of inflammatory cytokines after intravenous injection and upon 660 nm laser irradiation. Finally, the assistance of the anti‐PD‐1 enhanced the infiltration of CTLs in tumor tissues and increased the secretion of immune‐related cytokines to fight against tumors to a higher level.^[^ ^155^ ^]^ Therefore, PDT combined with chemotherapy may enhance the ICD stimulation to activate T lymphocytes for immune‐mediated tumor eradication.

### 5.6 MPNs for Combinational Immunotherapy with Other Therapies

SDT is a recently developed therapeutic modality that combines sonosensitizers with ultrasound to enhance the production of ROS to kill cancer cells. Some studies have shown that SDT has tremendous potential to activate adaptive antitumor immunity through the SDT‐mediated ICD effect.^[^ ^166^ ^]^ In 2022, Tian et al. conducted a phenolic nanoadjuvant developed by self‐assembly of the sonosensitizer polymer (PEG‐*b*‐IR), GSH inhibitor (sabutoclax), Mn^2+^, and TME acidic sensitive phenolic polymer (PEG‐*b*‐Pho) via metal phenolic coordination. The combinational action of SDT‐mediated ICD effect and Mn^2+^ promoted the activation of the cGAS–STING pathway and significantly enhanced DC maturation. The MPNs sensitize tumors to PD‐L1, resulting in efficiently inhibiting the growth of distant tumors and restraining lung metastasis.^[^ ^156^ ^]^

Irreversible electroporation (IRE) is a nonthermal ablation technique that employs brief high‐voltage electrical pulses to permanently disrupt cell membranes and induce cell death, which is a novel ablative technique used for clinical treatment.^[^ ^167^ ^]^ In 2022, Han et al. designed the MPN nanoparticles in combination with IRE, which are synthesized by coordinating TA with Mn^2+^, and subsequent coating with CpG–ODNs via hydrogen bonding for cancer immunotherapy. IRE could induce ICD and release TAAs and DAMPs, and CpG–ODN‐coated Mn–PN nanoparticles could induce M1 polarization by internalization in macrophages.^[^ ^157^ ^]^

## 6 MONs for Cancer Immunotherapy

MSNs have been identified as promising antitumor immunoadjuvant as a classic type of delivery platform, which have attracted features including large specific surface area, adjustable particle size, easy surface functionalization, tunable pore size, and inherent biocompatibility.^[^ ^168^ ^]^ However, the nondegradability of MSNs stemming from the inert Si—O—Si framework may cause trouble in vivo degradation and long‐term biosafety.^[^ ^34^ ^]^ In recent years, incorporating hydrophobic organic functional groups in the framework of MONs is an attractive strategy that can change the Si—O—Si framework and promote degradation under physiological conditions.^[^ ^169^ ^]^ MONs are inorganic and organic hybrid nanomaterials with controllable degradation, high tumor accumulation, and high biocompatibility. Moreover, the hybridization of diverse organic groups will endow MONs with a variety of functionalities. Therefore, MONs can be used as a vaccine delivery platform to provide tumor antigens directly or as a vaccine adjuvant. They can also synergize with other combinational immunotherapies such as PDT, PTT, and SDT to kill cancer cells immunologically and release tumor antigens in situ to enhance cancer immunotherapy. **Table** **5** summarizes the MONs used for cancer immunotherapy.

**Table 5:** Summary of the research on MONs for cancer immunotherapy

Type	Diameter	Doping	Payloads/surface modification	Administration route	Tumor model	Ref.
Vaccine	≈200 nm	Ethane	OVA	s.c.	B16F10	[170]
Vaccine	200 nm	Tetrasulfide	OVA, CpG/PEI	s.c.	B16‐OVA	[171]
Vaccine	110 nm	Ethane	OVA (iron oxide‐embedded)	i.t.	EG7‐OVA	[172]
Vaccine	110 nm	Benzene, ethylene	OVA, CpG	s.c.	B16‐OVA	[173]
Vaccine	95 nm	Diselenide	Annexin A5/hyaluronate‐modified bacterial outer membrane vesicles	i.v.	4T1	[174]
Drug delivery	≈200 nm	Cu^2+^, tetrasulfide	DOX	i.t.	4T1	[175]
Drug delivery	165 nm	Disulfide	Curcumin, iron oxide nanoparticles	i.t.	4T1	[176]
Drug delivery	≈200 nm	Disulfide	HCPT, siRNA/BSA, PEI, PEG	i.v.	B16F10, 4T1	[177]
Drug delivery	140 nm	Disulfide	Anti‐PD‐L1, indoximod/annexin A1 antibody	i.v.	4T1	[178]
Drug delivery	65 nm	Diselenide	KP1339	i.t.	4T1	[179]
Drug delivery	60 nm	Diselenide	DOX/cancer‐cell‐derived membrane fragments	i.v.	4T1	[180]
Drug delivery	≈200 nm	Ethane	DOX/TRAIL	i.v.	MCF‐7	[181]
PDT	250 nm × 100 nm	Disulfide (Janus magnetic MONs)	Ce6/breast cancer cell membrane	i.v.	MCF‐7	[182]
PDT	73 nm	Diselenide	Methylene blue, DOX/PEG	i.v.	4T1	[183]
PTT	80 nm	Diselenide	DOX/PEI, ICG hybrid *N*‐isopropyl acrylamide	i.v.	4T1	[184]
IRE	40–50 nm	Disulfide	TGF‐*β* inhibitor (SB525334)	i.t.	Panc02	[167]

### 6.1 MONs for Vaccine Delivery

Various nanomaterial delivery systems have been designed to develop more efficient and safer vaccines. Recent studies have demonstrated that novel nanoparticles significantly improve the immunogenicity of vaccines due to their intrinsic immunostimulatory properties.^[^ ^185^ ^]^ The hydrophobicity of nanoparticle‐based vaccine adjuvants can help to control the interaction between the encapsulated antigens or nanoparticles with immune cells.^[^ ^186^ ^]^

**Figure 10:**
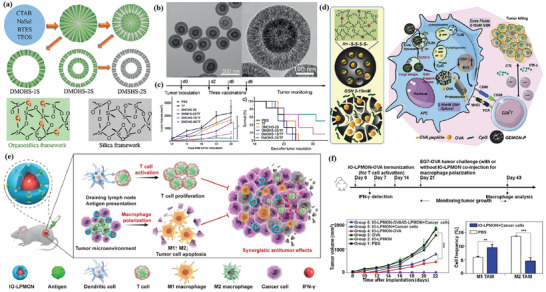
MONs as cancer vaccine delivering antigens and/or adjuvants. a) Schematic illustration of the synthesis procedure of dendritic mesoporous organosilica spheres with controllable number (n) of shells (DMOHS‐nS) and double‐shelled dendritic mesoporous silica spheres (DMSHS‐2S). b) Transmission electron microscopy (TEM) images of DMON‐2S. c) Antitumor performance of various vaccine formulations. Reproduced with permission.^[^ ^170^ ^]^ Copyright 2017, Wiley‐VCH. d) Schematic illustration of GDMON–P + OVA + CpG‐enhanced cancer immunotherapy. Reproduced with permission.^[^ ^171^ ^]^ Copyright 2018, Elsevier Ltd. e) Schematic illustration of the simultaneous T‐cell activation and macrophage polarization for potent antitumor immunotherapy using the IO–LPMONs. f) IO–LPMONs inhibit tumor growth by causing T‐cell activation and M1 polarization in vivo. Reproduced with permission.^[^ ^172^ ^]^ Copyright 2019, Wiley‐VCH.

Therefore, MONs with hydrophobic organic groups have the potential adjuvant property of stimulating antitumor immunity. In 2017, Yang et al. developed a hollow multishelled dendritic mesoporous organosilica encapsulating OVA (**Figure** **10**a,b).^[^ ^170^ ^]^ Compared to single‐shelled structure or pure silica, the double‐shelled dendritic structure (DMON‐2S) is beneficial for high payload and sustained release, and the organosilica framework with hydrophobicity improves the interaction of nanoparticles with APCs and induces endolysosomal escape and provide superior immunity in cancer immunotherapy (Figure 10c). Studies have shown that ROS can facilitate immune responses, especially for the activation of DCs,^[^ ^187^ ^]^ but the defense system of cells uses intracellular antioxidant GSH to neutralize excessive cytotoxic ROS. Lu et al. reported GSH‐depletion dendritic MONs (GDMON) with tetrasulfide bond in the framework as a self‐adjuvant and codelivery nanocarrier (Figure 10d).^[^ ^171^ ^]^ GDMON is capable of OVA and TLR9 agonist CpG into APCs. The reaction between GSH and tetrasulfide bond diminishes the intracellular GSH level, causing increased ROS level and the release of OVA and CpG to stimulate immune activity.

As the primary component of the innate immune system, TAMs can be polarized from immunosuppressive M2 phenotype toward tumoricidal M1 phenotype to improve tumor immunotherapeutic efficacy.^[^ ^188^ ^]^ Chen et al. designed iron‐oxide‐embedded large‐pore MONs (IO–LPMONs) to encapsulate OVA. IO–LPMONs deliver OVA to DCs and activate DCs.^[^ ^172^ ^]^ Moreover, IO–LPMONs also act as an immune modulator to polarize TAMs from immunosuppressive M2 to M1 phenotype, which induces apoptosis of tumor cells (Figure 10e,f). Otherwise, a type of benzene‐bridged MONs was explored as a novel immunoadjuvant and codelivery platform for both antigen and TLR9 agonists, which stimulate the maturation of DCs and efficiently coencapsulate OVA and CpG into immune cells, leading to 100% tumor‐free mice in 25 days in aggressive OVA‐expressed B16F10 melanoma model.^[^ ^173^ ^]^ Li et al. presented a strategy that exploits the primary tumor as antigens to initiate personalized antitumor immunity.^[^ ^174^ ^]^ They designed a diselenide‐bridged hollow mesoporous organosilica nanoparticle to immobilize protein annexin A5 (ANX5). The diselenide bond would be degraded under the oxidation tumor microenvironment or reductive intracellular environments and then released ANX5 to bind phagocytic marker PS. The recognition of apoptotic cells by macrophages is inhibited. Then apoptotic cells will undergo secondary necrosis and release tumour‐associated antigen epitopes (TAEs) and DAMPs to reinvigorate antitumor immune responses, which led to complete tumor eradication in about 50% of mice with orthotopic breast tumors and generated long‐lasting immunological memory.

### 6.2 MONs for Drug Delivery

**Figure 11:**
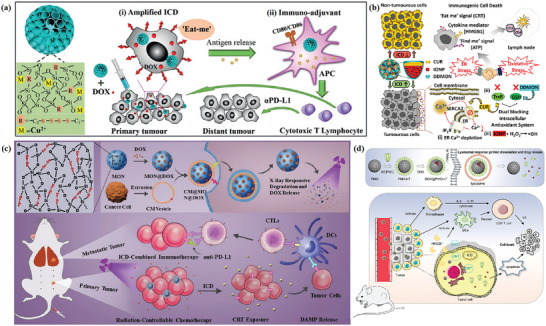
MONs for drug delivery. a) The nanostructure and composition of mesoporous organosilica nanoparticles. After mixing with DOX for local administration, the nanoreactors are able to i) amplify ICD, ii) act as immune adjuvants to stimulate the maturation of immune cells. Reproduced with permission.^[^ ^175^ ^]^ Copyright 2018, Wiley‐VCH. b) Illustration of the DDMON–IONP–CUR ICD nanoinducer for cancer immunotherapy. Reproduced with permission.^[^ ^176^ ^]^ Copyright 2020, American Chemical Society. c) Schematic of synthesis of diselenide‐bond‐bridged MONs for low‐dose X‐ray‐radiation‐controllable drug release. Reproduced with permission.^[^ ^180^ ^]^ Copyright 2020, Wiley‐VCH. d) DOX@PMO–hT (HA5ST modified with three amino acids Pro‐Lys‐Cys at the C‐terminus, hT) synthesis and antitumor mechanisms. Reproduced with permission.^[^ ^181^ ^]^ Copyright 2022, Elsevier.

Chemotherapy is limited by off‐target toxicity and ineffectiveness against distant tumors. Therefore, amplifying the chemotherapy‐driven ICD effect for safe and efficient cancer chemo‐immunotherapy remains a challenge. Biodegradable MONs are hopeful for anticancer drug delivery. In 2018, Yang et al. designed a large‐pore dendritic hybrid silica framework incorporated with Fenton's reagents (Cu^2+^) and tetrasulfide groups to amplify the DOX‐mediated ICD effect (**Figure** **11**a).^[^ ^175^ ^]^ Cu^2+^ produces cytotoxic ROS and tetrasulfide groups consume the antioxidant GSH, resulting in elevated intracellular oxidative stress to improve ICD. At last, the nanoreactors exhibited excellent anticancer performance for both primary and distant tumors when forming synergism with ICB. Moreover, Dai et al. presented a similar ICD nanoinducer (DDMON–CUR–IONP) composed of disulfide‐bond‐incorporated organosilica nanoparticles, CUR, and iron oxide nanoparticles, which can deplete intracellular glutathione, produce hydroxyl radicals, and induce cancer‐cell‐specific Ca^2+^ depletion as well as thioredoxin reductase inhibition (Figure 11b).^[^ ^176^ ^]^ Compared to conventional molecular ICD inducers, the simultaneous enhancement of intracellular ER stress and oxidative stress could induce strong ICD and anticancer activity. As the product of aerobic glycolysis in tumors, lactate could regulate the immunosuppressive TME.^[^ ^189^ ^]^ Li et al. combined inhibiting lactate efflux and chemotherapy to remove immunosuppressive TME.^[^ ^177^ ^]^ They synthesized a GSH‐responsive hollow mesoporous‐silica‐based nanoparticles to load hydroxycamptothecin (HCPT) drug and further immobilized bovine serum albumin (BSA) onto its surface for effective encapsulation and then coupled with monocarboxylate transporter 4 (MCT‐4)‐inhibiting siRNA to inhibit lactate efflux. Tumor vasculature significantly hampers the efficient delivery of nanomedicine into tumors.^[^ ^190^ ^]^ Huang et al. developed an annexin‐A1‐antibody‐installed MONs carrying anti‐PD‐L1 antibody and IDO inhibitor (indoximod), which can target luminal endothelial cells and efficiently enhance nanomedicine extravasation via the transendothelial delivery route, thus distinctly reverse the immunosuppressive TME.^[^ ^178^ ^]^ Otherwise, Zhang et al. designed diselenide‐bridged MONs coated with chemotherapeutic ruthenium compound (KP1339).^[^ ^179^ ^]^ The controlled release of KP1339 was achieved by competitive coordination and matrix degradation in response to GSH. Moreover, MONs evoked ROS production and GSH depletion, leading to ER stress, thus amplifying the ICD effect of KP1339 and boosting robust antitumor immunological responses. Drug carriers that respond to external stimuli such as X‐ray radiation are also promising. Shao et al. designed cancer‐cell‐membrane‐coated MONs containing X‐ray and ROS‐responsive diselenide bonds encapsulated with DOX for chemo‐immunotherapy (Figure 11c).^[^ ^180^ ^]^ The diselenide bond is sensitive to X‐ray radiation, which promotes the release of DOX and enhances the ICD effect. In 2022, Feng et al. designed periodic mesoporous organosilica nanoparticles (PMOs) decorated with tumor‐necrosis‐factor‐related apoptosis‐inducing ligand (TRAIL) and loaded with DOX (Figure 11d). TRAIL enhanced the nanoparticle‐targeting capacity and worked synergistically with DOX against breast cancer cells.^[^ ^181^ ^]^

### 6.3 MONs for Combinational Immunotherapy with Other Therapies

**Figure 12:**
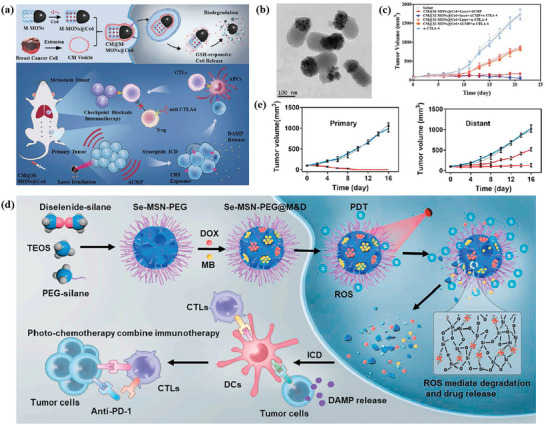
a) Schematic illustration of the synthetic procedure for the cancer‐cell‐membrane‐cloaked Ce6‐loaded Janus magnetic mesoporous organosilica nanoparticles (CM@M‐MON@Ce6) and their application for combined PDT and magnetic hyperthermia to further potentiate a CTLA‐4 blockade to enhance synergistic antitumor immunity in combating cancer metastasis. b) TEM images of CM@M‐MON@Ce6. c) The synergistic effects of CM@M‐MON@Ce6‐mediated PDT and magnetic hyperthermia in combination with anti‐CTLA4 checkpoint blockade. Reproduced with permission.^[^ ^182^ ^]^ Copyright 2019, Wiley‐VCH. d) Schematic of the synthetic procedure of Se–MSN–PEG with cascading drug release and amplifying ICD manners and their application for efficient and safe cancer chemo‐photo‐immunotherapy. e) Antitumor performance of Se–MSN–PEG in 4T1 bilateral tumor model. Reproduced with permission.^[^ ^183^ ^]^ Copyright 2022, Elsevier.

Stimuli‐responsive drug delivery systems have been extensively investigated, especially for light irradiation. In 2019, Wang et al. reported nanoparticles mediated combination of PDT and magnetic hyperthermia to enhance immunotherapy. Janus nanobullets combine disulfide‐bridged MONs bodies with magnetic heads (M‐MONs) embedded with Ce6 to achieve tumor microenvironment (dual redox/pH)‐responsive drug release (**Figure** **12**a,b). Combined with anti‐CTLA‐4 antibodies, the nanoparticles could eradicate primary and deeply metastatic tumors (Figure 12c).^[^ ^182^ ^]^ In 2022, Yang et al. designed a polyethylene‐glycol‐modified, diselenide‐bridged MON coated with DOX and photosensitizer methylene blue (Figure 12d).^[^ ^183^ ^]^ Upon low‐dose red light irradiation, ROS mediates the diselenide bond cleavage resulting in the degradation of organosilica and drug release. The cascade chemo‐PDT boosts the ICD effect and robust antitumor immunity responses in the 4T1 bilateral tumor model (Figure 12e). Moreover, PTT can also improve tumor accumulation and the ICD effect. In 2022, Peng et al. designed a core–shell nanotransformer loaded with DOX that integrates diselenide‐bridged MONs as ROS‐responsive core with an ICG‐hybrid *N*‐isopropyl acrylamide layer as a thermosensitive shell.^[^ ^184^ ^]^ Upon NIR light irradiation, the photothermal effects facilitate the dissociation of the thermosensitive shell and ROS cleaves the diselenide bond of MONs to release DOX. When combined with anti‐PD‐1, the nanotransformer remarkably blocks primary tumor growth of breast cancer. Using glutathione‐responsive degradable MSNs loaded with SB525334, an inhibitor of the transforming growth factor‐*β*1 (TGF‐*β*1) receptor, it is demonstrated that local inhibition of TGF‐*β* promotes neutrophil polarization into an antitumor phenotype, which enhances pancreatic cancer response to combined IRE and anti‐PD‐1.^[^ ^167^ ^]^

## 7 Metallofullerene Nanomaterials for Cancer Immunotherapy

As a unique class of carbon allotropes, fullerenes have been studied intensively since their discovery. Derived from the unique structures of the outer fullerene cages and encaged metal clusters, metallofullerenes possess excellent stability, paramagnetism, large surface area, and a unique surface that is easy to be functionalized.^[^ ^191^ ^]^ Gadofullerenes with definite structures, low toxicity, and easy excretion from the body are widely applied in the biomedicine field. Gd@C_82_(OH)*_x_* is a C_82_ fullerene derivative with a gadolinium atom entrapped in the core of the carbon cage and its surface is modified with *x* number of hydroxyl groups. Gd@C_82_(OH)*_x_* congregates into nanoparticles through large molecular interactions in aqueous solutions, and the surface of nanoparticles is usually embraced with water molecules through hydrogen bonds, which allow the nanoparticles to have good biocompatibility in vivo.^[^ ^192^ ^]^ In addition, Gd@C_82_(OH)*_x_* was studied originally as a new generation of MRI contrast agent due to its high proton relaxivity.^[^ ^193^ ^]^ **Table** **6** summarized the gadofullerene nanomaterials used for cancer immunotherapy.

**Table 6:** Summary of the research on metallofullerenes for cancer immunotherapy

Material type	Diameter	Surface modification	Administration route	Tumor model	Key findings	Ref.
[Gd@C_82_(OH)_22_]*_n_*	22 nm	Hydroxyl	i.p.	H22 hepatoma	Improve immunity and cause no damage	[192]
[Gd@C_82_(OH)_22_]*_n_*	92 nm	Hydroxyl	i.p.	LLC	Stimulated T cells and macrophages to release TNF‐*α* (Th1)	[194]
[Gd@C_82_(OH)_22_]*_n_*	25 nm	Hydroxyl	–	–	Potent activator of DCs and Th1 immune responses	[195]
C_60_(OH)_22_, Gd@C_82_(OH)_22_	180, 122 nm	Hydroxyl	i.v. Macrophages activated by C60(OH)_22_ or Gd@C_82_(OH)_22_ nanoparticles	B16	Activate macrophages via NF‐*κ*B pathway adoptive immunotherapy	[196]
Gd@C_82_–Ala	130 nm	*β*‐alanines	i.v.	4T1‐Luc	Surgery with PDT to produce ROS and enhance DC maturation	[197]
Gd@C_82_–Ala	68 nm	*β*‐alanines	i.p.	4T1	Reprogram TAMs to M1‐like and promote ICB efficacy	[35]

**Figure 13:**
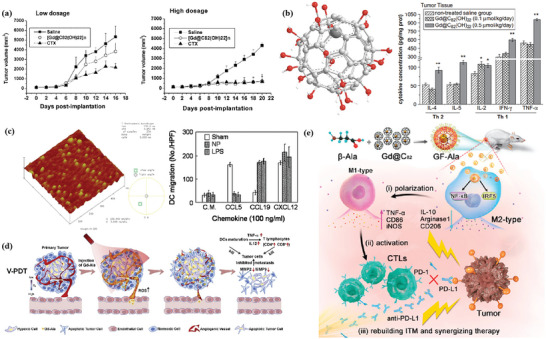
a) Growth inhibitory curves of the treatment with 22 nm [Gd@C_82_(OH)_22_]*_n_* nanoparticles on murine H22 hepatoma in low and higher dosage. Reproduced with permission.^[^ ^192^ ^]^ Copyright 2005, American Chemical Society. b) Structure of Gd@C_82_(OH)_22_ nanoparticles and IL‐2, IL‐4, IL‐5, TNF‐*α*, and IFN‐*γ* cytokine expression levels in mouse tumor tissues. Reproduced with permission.^[^ ^194^ ^]^ Copyright 2009, Elsevier. c) AFM image of the [Gd@C_82_(OH)_22_]*_n_* nanoparticles and DCs, responsiveness to selected chemokines. Reproduced with permission.^[^ ^195^ ^]^ Copyright 2010, American Chemical Society. d) Schematic representation of V‐PDT based on Gd@C_82_–Ala. Reproduced with permission.^[^ ^197^ ^]^ Copyright 2019, Elsevier. e) Process of Gd@C_82_–Ala in vivo eliciting immune responses by reprogramming TAMs. Reproduced with permission.^[^ ^35^ ^]^ Copyright 2020, American Chemical Society.

In 2005, Gd@C_82_(OH)_22_ nanoparticles were shown to have antitumor effects. Hematoxylin‐eosin (HE)‐stained tumor tissues showed that the nanoparticles could improve immunity and interfere with tumor invasion, suggesting that they may upregulate the immune system.^[^ ^192^ ^]^ Notably, compared to cyclophosphamide and cisplatin, [Gd@C_82_(OH)_22_]*_n_* particles cause no observable damage to important organs and have higher antineoplastic efficiency (**Figure** **13**a). Then, they further studied the effect of Gd@C_82_(OH)_22_ nanoparticles on the immune system of LLC tumor‐bearing mice and found that the nanoparticles could stimulate T cells and macrophages to release significantly greater quantities of tumor necrosis factor alpha (TNF‐*α*), which play a crucial role in Th1 cellular immune processes (Figure 13b).^[^ ^194^ ^]^ In 2010, Yang et al. found that [Gd@C_82_(OH)_22_]*_n_* can induce DCs to become functionally mature by stimulating DC production of cytokines including IL‐12p70, upregulating DC costimulatory and MHC molecules, and switching DCs from a C‐C motif chemokine ligand (CCL) 5‐responsive to a CCL19‐responsive phenotype, which enhances antigen‐specific Th1 immune response, suggesting a novel basis for its potent antitumor activity (Figure 13c).^[^ ^195^ ^]^ In 2016, Tang et al. investigated the ability of polyhydroxylated fullerenes (C_60_(OH)_22_ and Gd@C_82_(OH)_22_ nanoparticles) to regulate macrophages for cancer immunotherapy. Furthermore, they back‐transfused macrophages that were activated by C_60_(OH)_22_ and Gd@C_82_(OH)_22_ nanoparticles to inhibit tumor metastasis and decrease the toxicity of traditional adoptive immunotherapy and found that nanoparticles both activated macrophages to secrete cytokines via the nuclear factor‐*κ*B (NF‐*κ*B) signaling pathway.^[^ ^196^ ^]^

Gadofullerene was commonly modified by various functional groups to be applied in vivo or provide unique properties. In 2019, Guan et al. reported the phototriggered tumor vascular therapy (V‐PDT) nanogadofullerene Gd@C_82_–Ala, which were intravenously injected and simultaneously subjected to light irradiation (Figure 13d).^[^ ^197^ ^]^ The activated Gd@C_82_–Ala could induce malignant tumor vascular disruption, where oxygen in blood vessels was employed efficiently to produce cytotoxic ROS. Gd@C_82_–Ala could also enhance DC maturation, further secreting TNF‐*α* and IL‐12, and then activated T lymphocytes. In 2020, Gd@C_82_ nanoparticles modified with *β*‐alanines were demonstrated to reprogram TAMs from tumor‐promoting M2 phenotype to tumoricidal M1 phenotype and increase the infiltration of CTLs, triggering effective antitumor immunity and achieving effective inhibition of tumor growth (Figure 13e).^[^ ^35^ ^]^ Notably, the modulation of the immunosuppressive tumor microenvironment by Gd@C_82_–Ala promotes the anticancer efficacy of anti‐PD‐L1 immune checkpoint inhibitor, achieving superior synergistic treatment. To conclude, exploring new biomedical applications of gadofullerene provides the carbon nanomaterial candidate for cancer immunotherapy.

## 8 Polymer–Lipid Hybrid Nanomaterials for Cancer Immunotherapy

Polymer–lipid hybrid nanoparticles are nanocarriers composed of an internal polymeric core enclosed by an outer lipid shell composed of one or more layers, which has the competence to encapsulate different types of payloads, no matter hydrophilic and hydrophobic drugs.^[^ ^198^ ^]^ **Table** **7** summarizes the polymer–lipid hybrid nanomaterials used for cancer immunotherapy.

**Table 7:** Summary of the research on polymer–lipid hybrid nanomaterials for cancer immunotherapy

Material type^a)^	Polymer (metal)/lipid	Diameter	Payloads	Surface modification	Administration route	Tumor model	Ref.
P–L	PLGA/DSPE–PEG, 1,2‐dipalmitoyl‐sn‐glycero‐3‐phosphoethanolamine (DPPC)	110 nm	Imatinib	tLyp1 peptide	i.v.	B16/BL6	[199]
P–L	PEGylated derivative of histidylated polylysine (PEG–HpK)/TriMan‐liposome	230 nm	OVA/E7 mRNA	–	i.v.	TC‐1, B16	[49]
P–L	PLGA/cholesterol, 1,2‐dioleoyl‐sn‐glycero‐3‐phosphocholine (DOPC), DSPE–PEG	119 nm	DNIC, DOX/TRAIL	–	i.v.	HCC	[200]
P–L	TK–PPE/DSPE–PEG, lecithin	91 nm	DOX, Ce6	–	i.v.	4T1	[201]
P–L	PCL–PEG–PCL/DOTAP	221 nm	Imiquimod, MPLA, OVA	Mannose	s.c.	E.G7‐OVA	[38]
P–L	PLGA/DSPE–PEG, DPPC	181 nm	Tin mesoporphyrin (SnMP)	scFv‐monomeric avidin fusion (sFVA), biotin, PEG	i.v.	U937	[202]
P–L	Polyethylene glycol‐b‐poly(aminoethyl methacrylate)‐b‐poly(2‐(diisopropyl amino) ethyl methacrylate (PEAD)/phosphatidyl choline, cholesterol	135 nm	DOX, siRNA of PD‐L1	–	p.t.	B16	[203]
P–L	PLGA/lecithin– tristearin	100–110 nm	Etoposide, siCD47	–	i.v.	B16F10	[198]
P–L	MGluPG–liposomes and lipoplexes	219 nm	Antigen and IFN‐*γ* gene	–	s.c.	E.G7‐OVA	[204]
M–L	Zinc phosphate NPs/dioleoyl phosphatidic acid (DOPA), DOPC, cholesterol, DSPE–PEG, MPLA	25–30 nm	Antigenic peptides (TRP2 and HGP100), MPLA	–	s.c.	B16F10	[205]
M–L	Zinc phosphate NPs/DOPA, DOPC, cholesterol, DSPE–PEG, MPLA	25–30 nm	B16F10‐derived tumor lysate, MPLA	–	s.c.	B16F10	[206]
M–L	Au^3+^/1,2‐dioleoyl‐sn‐glycero‐3‐ethylphosphocholine (EDOPC), DC–Chol, 1,2‐diphytanoyl‐sn‐glycero‐3‐phosphoethanolamine (DPhPE)	113 nm	TGF‐*β* pC9sTgf	–	i.t.	B16F10	[207]

^a)^ P–L: polymer–lipid hybrid nanomaterials, M–L: metal–lipid hybrid nanomaterials.

**Figure 14:**
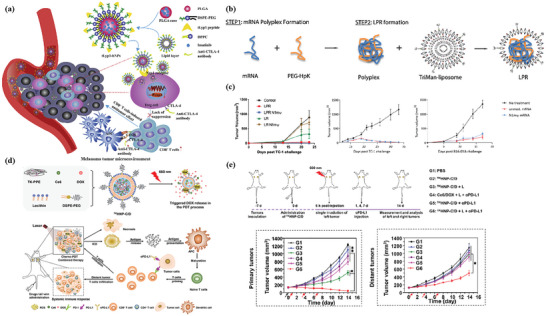
a) Schematic illustration of tLyp1‐hNPs. Reproduced with permission.^[^ ^199^ ^]^ Copyright 2018, Elsevier. b) Synthesis of LPR nanoparticles. c) Tumor growth curves of TC‐1‐ and B16‐OVA‐inoculated mice treated as depicted by i.v. immunization. Reproduced with permission.^[^ ^49^ ^]^ Copyright 2018, American Chemical Society. d) Preparation and characterization of ROS‐sensitive hybrid nanoparticle encapsulated Ce6 and DOX with neutral lipid lecithin and DSPE‐PEG as the outer shell and homopolymer poly(thioketal phosphoe) (TK‐PPE) as the hydrophobic core (^TK^HNP‐C/D). e) The potentiated checkpoint blockade immunotherapy by ^TK^HNP‐C/D‐mediated chemo‐PDT and aPD‐L1 in a bilateral 4T1 tumor model. Reproduced with permission.^[^ ^201^ ^]^ Copyright 2019, Elsevier.

Treg cells are a type of CD4^+^ T cells that can downregulate the activation and proliferation of a wide range of immune cells.^[^ ^208^ ^]^ In 2018, Ou et al. developed tLyp1‐peptide‐conjugated polymer–lipid hybrid nanoparticles for targeting the Nrp1 receptor on Treg cells in the tumor microenvironment (**Figure** **14**a).^[^ ^199^ ^]^ The encapsulation of Treg cell inhibition drug Imatinib in tLyp1‐hybrid NPs (hNPs) impedes the suppressive function of Treg cells via blocking signal transducer and activator of transcription 3 (STAT3) and signal transducer and activator of transcription 5 (STAT5) signaling, and the system can help bolster T‐cell responses with the combination of anti‐CTLA4 immunocheckpoint blockade. Lipid‐based messenger RNA (mRNA) nanocomplexes have emerged as a powerful approach to promote potent antitumor T‐cell responses,^[^ ^209^ ^]^ but nanoparticles coated with mRNA may cause an increased risk of adverse events. Therefore, polymer–lipid hybrid nanoparticles (LPRs) are also used to deliver mRNA to avoid inflammatory responses and hematological toxicities. Van der Jeught et al. explored hybrid lipid‐shell polymer‐core mRNA nanoparticles to improve colloidal stability and reduce cytotoxicity. LPR reduces inflammatory responses without hampering T‐cell immunity and showed superior effectiveness in controlling tumor growth compared to lipid‐based mRNA nanoparticles (Figure 14b,c).^[^ ^49^ ^]^ In addition, polymer–lipid‐hybrid‐nanomaterials‐mediated low‐dose nitric oxide (NO) delivery was also reported to normalize tumor vessels and reprograms the immunosuppressive TME to immunostimulatory phenotype.^[^ ^200^ ^]^ The NO‐delivery system was assembled by the encapsulation of a [Fe(µ‐SEt)_2_(NO)_4_] (DNIC) into lipid–PLGA nanoparticles. The release of NO normalizes tumor vessels, and enhances the delivery of small‐molecule anticancer drugs and macromolecular therapeutics to suppress tumor progression in combination with immunotherapy. Hu et al. reported ROS‐sensitive lipid–polymer hybrid nanoparticles using DOX‐based chemotherapy and Ce6‐based PDT to potentiate the antitumor efficacy of anti‐PD‐L1. The nanoparticles were composed of neutral lipid lecithin and distearoylphosphatidylethanolamine (DSPE)–PEG as the outer shell and ROS‐sensitive homopolymer poly(thioketal phosphoester) (TK‐PPE) as the hydrophobic core to stabilize the nanoparticles and efficiently encapsulate Ce6 and DOX (Figure 14d,e).^[^ ^201^ ^]^ Zhang et al. constructed a mannose‐targeting lipid‐polymer hybrid nanoparticle (LPNP) vaccine system from biodegradable polymer poly(epsilon‐caprolactone) (PCL)–PEG–PCL and cationic lipid 1,2‐dioleoyl‐3‐trimethylammonium‐propane (DOTAP) for a programmed codelivery of an antigen and dual TLR agonists. The sponge effect of DOTAP can facilitate the endosomal escape of antigens for cross‐presentation by DCs.^[^ ^38^ ^]^ In 2020, Yong et al. constructed lipid–polymer hybrid nanoparticles loaded with HO1 inhibitor tin mesoporphyrin (SnMP) and noncovalently modified with an engineered single chain antibody (T‐hNP) for active targeting of acute myeloid leukemia cells and passive targeting to leukemia‐associated myeloid cell.^[^ ^202^ ^]^ In addition, Wang et al. developed cationic polymer–lipid hybrid nanovesicles as a delivery system for DOX and PD‐L1 siRNA. As a result, PD‐L1 blockade synergized with DOX triggered robust therapeutic antitumor T‐cell responses and eradicated tumors in 30% of mice bearing B16 melanoma.^[^ ^203^ ^]^ In 2021, Abdel‐Bar et al. investigated lipid–polymer hybrid nanoparticles (LPHs) as a platform for the delivery of siRNA and etoposide, and two strategies were attempted to introduce siRNA into the formulation. Next, the ability of optimized LPH formulations to improve siRNA uptake and gene silencing was investigated in mouse melanoma B16F10 cells.^[^ ^198^ ^]^ In addition, the ability to deliver contents into the cytosol via membrane fusion with the endosome of pH‐sensitive liposomes might be of particular importance.^[^ ^210^ ^]^ Yuba et al. designed 3‐methylglutarylated‐poly(glycidol) (MGluPG)‐modified liposomes and cationic liposomes, respectively, for the delivery of antigenic protein OVA and IFN‐*γ*‐encoding plasmid DNA and the two liposomes formed hybrid nanomaterials via electrostatic interactions.^[^ ^204^ ^]^

**Figure 15:**
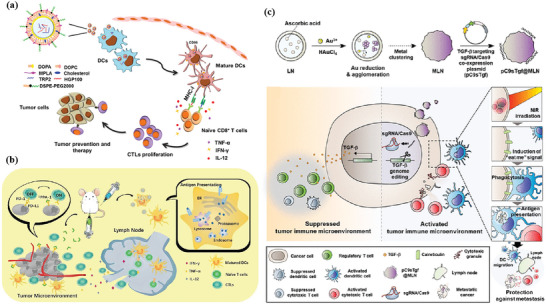
a) Scheme of antitumor immunity induced by LZnP‐NP‐based peptide vaccines. Reproduced with permission.^[^ ^205^ ^]^ Copyright 2015, Elsevier. b) Schematic illustration of TLS‐loaded LZnP nanovaccine combined with immune checkpoint antagonist ^d^PPA‐1 to induce antitumor immunity. Reproduced with permission.^[^ ^206^ ^]^ Copyright 2018, Wiley‐VCH. c) Construction of pC9sTgf@MLNs and proposed mechanisms for its restructuring of the tumor immune microenvironment. Reproduced with permission.^[^ ^207^ ^]^ Copyright 2021, American Chemical Society.

Lipid‐based hybrid nanoparticles with outstanding biocompatibility, biodegradability, low toxicity, and controllable size are also attractive for cancer immunotherapy. In 2016, Zhuang et al. developed lipid‐coated zinc phosphate hybrid nanoparticles via the properties and influence on the immune system of zinc for coencapsulating the antigenic peptides (TRP2 and HGP100) and monophosphoryl lipid A (MPLA, TLR4 agonist).^[^ ^205^ ^]^ It has been reported that zinc and phosphate are essential elements for life, and zinc can influence the immune system by promoting the activity of APCs.^[^ ^211^ ^]^ The lipid improved the stability and biocompatibility of nanoparticles and the entrapment capability of lipid adjuvant MPLA (**Figure** **15**a). And in 2019, they used lipid zinc phosphate hybrid nanoparticles to load MPLA‐ and B16F10‐melanoma‐cell‐derived tumor lysate for vaccination (Figure 15b).^[^ ^206^ ^]^ The immune checkpoint antagonist, d‐peptide antagonist (^D^PPA‐1), was also involved in treatment, which helped tumor lysate (TLS)‐loaded lipid‐enveloped zinc phosphate (LZnP) nanovaccine efficiently prime DCs and induced CTL response. In 2021, Kim et al. reported a metal‐lipid‐hybrid‐nanoparticle (MLN)‐mediated gene editing of TGF‐*β* to restructure the TME. In MLNs, Au metal was encapsulated to enable a photothermal effect to increase the exposure of DAMPs on tumor cells upon NIR irradiation. Intratumoral injection of nanoparticles and NIR irradiation resulted in the ablation of primary tumors in the B16F10 model (Figure 15c).^[^ ^207^ ^]^

## 9 Biomacromolecules‐Based Hybrid Nanomaterials for Cancer Immunotherapy

The surface of organic or inorganic nanomaterials is inevitably covered with various biomolecules forming a protein corona, which reduces the targeting ability of the nanomaterials to some extent. In comparison, biomacromolecules have good biocompatibility with surrounding normal tissues, which can be advantageous for clinical application.^[^ ^212^ ^]^ In this section, we focus on the important role of biomacromolecules hybrid nanomaterials in cancer immunotherapy, including membrane‐, protein‐, and nucleic‐acid‐based nanoparticles. **Table** **8** summarizes the biomacromolecules used for cancer immunotherapy.

**Table 8:** Summary of the research on biomacromolecule nanomaterials for cancer immunotherapy

Material type	Hybrid strategies	Diameter	Payloads/surface modification	Administration route	Tumor model	Key findings	Ref.
Membranes	RBC and PLT membrane	150 nm	Liposome, tirapazamine, Ce6	i.v.	B16F10	Reverse the drawbacks of SDT	[213]
	RBC and cancer cell membrane	200 nm	–	i.v.	B16F10‐Luc, 4T1‐Luc	Personalized vaccination combined with aPDL1 blockade	[39]
	RBC and ID8 membrane	≈50 nm	Fe_3_O_4_–ICG	i.t.	Bilateral flank tumor models (ID8/ID8, ID8/B16‐F10)	Synergistic PTT–immunotherapy for ovarian cancer	[40]
	DC and cancer cell membrane	146 nm	PCN‐224 MOF	s.c.	4T1	Combination of two pathways (direct T‐cell activation and indirect DC‐to‐T activation)	[214]
	C6 cell and DC membranes	154 nm	Docetaxel (DTX) nanosuspensions	i.v.	C6 glioma	Tumor‐targeted drug delivery combined with immunotherapy in gliomas	[215]
	Macrophages and cancer cell membrane	142 nm	PLGA, Met‐CO_2_, siFGL1	i.v.	4T1	Hybrid biomimetic membrane combined with Met‐FGL1 blockade	[216]
	PLTs, M1‐macrophage, and cancer cell nanovesicles	100 nm	2'3'‐cyclic‐GMP‐AMP (cGAMP)	i.v.	B16F10, 4T1	Block CD47–SIRP*α* signaling axis and promote M2‐to‐M1 repolarization	[217]
	Melanoma cytomembrane vesicles and attenuated *Salmonella* outer membrane vesicles	60–300 nm	PLGA–ICG NPs	Intradermal	B16F10, 4T1	Eukaryotic–prokaryotic vesicle nanoplatform combined with PTT	[218]
	Bacterial outer membrane vesicle and B16F10 cancer cell membrane	≈200 nm	HPDA NPs	i.v.	B16F10	OMV immunotherapy combined with HPDA‐mediated PTT	[219]
	Artificial liposomes and tumor‐derived nanovesicles	166 nm	DOX	s.c.	B16F10, LLC, 4T1	Combined chemotherapy with immunotherapy	[220]
	Macrophage and thylakoid membrane	259 nm	HCQ encapsulates mannose‐decorated HMPB	i.v.	4T1	Homing effect of macrophage membrane and TK membrane catalyzed H_2_O_2_ to generate O_2_ for hypoxia mitigation	[221]
Proteins	Fe–GA–BSA/OVA/HSA	194 nm	–	i.v.	4T1	Incorporate various functional proteins to form protein–Fe–GA nanonetworks with good biocompatibility	[150]
	Al, ZIF‐8–OVA	70 nm	CpG	s.c.	EG7‐OVA	Safe and effective cancer vaccines	[63]
	Al–BSA	25 nm	Ce6	i.v.	B16F10	Engineer photosensitizer and alumina adjuvant	[222]
	ZnS–BSA	≈100 nm	–	i.v.	HCC	Activation of cGAS–STING pathway and produce ROS	[223]
	CaCO_3_–OVA	≈500 nm	–	s.c.	EG7	High loading and endosome escape	[224]
	CaO_2_–OVA	≈80 nm	–	s.c.	B16‐OVA	Generate ROS leading to endo‐/lysosomal lipid peroxidation	[225]
	MnO_2_–Alb	56 nm	ICG, phenformin	i.v.	CT26	H_2_O_2_‐responsive	[226]
	Polymer–serum albumin (SA)	34 nm	–	i.v.	B16F10	Capture and remove galectin‐1 (Gal‐1)	[227]
Nucleic acid	DNA origami rectangle hybrid antigen (peptide) and TLR agonists (double‐stranded RNA (dsRNA) and CpG DNA)	≈19 nm × ≈90 nm	Antigen and adjuvant	s.c.	B16‐OVA	DNA locking strands outside the nanostructures open the vaccine in lysosomes	[228]
	Triangular DNA origami framework hybrid six pairs of digoxin molecule	120 nm	–	–	–	Capture transient conformations of IgGs	[229]
	PEI/DNA complexes hybrid live attenuated *Salmonella*	–	–	p.o.	B16F10	Nanoparticle‐coated bacteria as oral DNA vaccines	[230]
	Concatemer CpG analogs hybrid Mg_2_PPi	≈1000 nm	–	i.t.	B16F10	Mg_2_PPi renders hybrid nanovaccines (hNVs) resistant to nuclease degradation and thermal denaturation	[231]
	LSD1 siRNA hybrid 3pRNA	19 nm	MnO_2_ NPs/BSA	i.v.	4T1	Innate sensing of the hybrid RNA promotes DC maturation	[232]
	VEGF RNAi hybrid targeted M2peptide	23 nm	Au NPs	Intratracheal	A549 lung adenocarcinoma tumors	Silence VEGF combined with M2‐peptide‐based targeted TAMs	[233]

### 9.1 Membrane‐Based Nanomaterials

**Figure 16:**
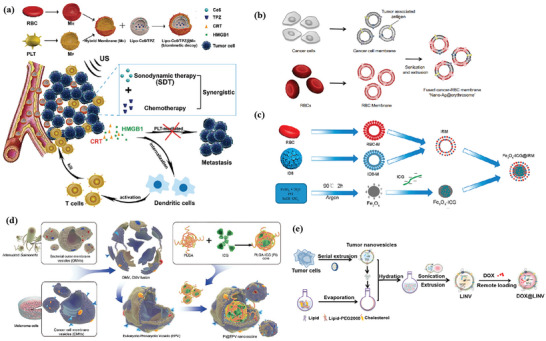
a) Schematic of the biomimetic decoy (Lipo–Ce6/TPZ@M_H_) preparation and synergistic SDT–chemotherapy leads to tumor ICD and DAMP release to elicit antitumor immunotherapy. Reproduced with permission.^[^ ^213^ ^]^ Copyright 2019, Wiley‐VCH. b) Schematic of preparation of nano‐Ag@erythrosomes by fusing tumor‐antigen‐associated cell membrane into nanoerythrosomes. Reproduced with permission.^[^ ^39^ ^]^ Copyright 2019, American Association for the Advancement of Science. c) Preparation of Fe_3_O_4_–ICG@IRM nanoparticles. Reproduced with permission.^[^ ^40^ ^]^ Copyright 2021, American Chemical Society. d) Schematic illustration of fabrication of eukaryotic–prokaryotic‐vesicle‐coated PI@EPV (poly(lactic‐co‐glycolic acid)‐ICG moiety, PI) nanovaccine. Reproduced with permission.^[^ ^218^ ^]^ Copyright 2020, Wiley‐VCH. e) Preparation of DOX@LINV by the confusion of tumor‐derived nanovesicles (TNVs) with artificial liposomes. Reproduced with permission.^[^ ^220^ ^]^ Copyright 2021, American Chemical Society.

In recent years, cell‐membrane‐based biomimetic nanoparticles have been in the spotlight of cancer immunotherapy. The nanomaterials camouflaged with cell membranes from red blood cells (RBCs), immune cells, and cancer cells possess the components of the natural cell membrane, which might provide the NPs with many desirable biological functions. Compared with the single cell membrane, hybrid membranes can endow more multifunctional and complex functions. A RBC and platelet (PLT) hybrid‐membrane‐camouflaged pH‐sensitive liposome loaded with sonosensitizer Ce6 and hypoxia‐activated TPZ is constructed to enhance SDT.^[^ ^213^ ^]^ The hybrid membrane retains PLT binding functions but is incapable of platelet‐mediated metastasis, and RBCs can prolong blood circulation lifetime. It has been indicated that HMGB1 is the key factor that interacts with TLR4 on PLTs and mediates PLT tumor cell interaction, which contributes to metastasis of melanoma.^[^ ^234^ ^]^ Hence, PLT membrane maintains binding interaction with high mobility group box protein 1 (HMGB1) induced by SDT–chemotherapy, preventing tumor extravasation and metastasis (**Figure** **16**a). In addition, tumor antigen recognition and phagocytosis from cancer cell membranes are important for immunotherapy. In 2019, Han et al. fused RBC membranes and tumor cell membranes loaded with tumor antigens to form nano‐Ag@erythrosomes for antigen delivery to the spleen to enhance cancer immunotherapy (Figure 16b).^[^ ^39^ ^]^ In 2021, Xiong et al. designed a hybrid biomimetic‐membrane‐fused murine‐derived ID8 ovarian cancer cell membrane with RBC membrane (IRM) to camouflage ICG‐loaded magnetic nanoparticles (Fe_3_O_4_–ICG@IRM) for PTT of ovarian cancer (Figure 16c).^[^ ^40^ ^]^ The ID8 and RBC cell membrane proteins make the nanoparticles exhibit specific self‐recognition of ID8 cells and prolonged circulation lifetime in blood to enhance PTT in ID8 tumors and induce the release of tumor antigens. Currently, immune cell membranes are also employed as ingredients for fusion with cytomembrane to endow the hybrid materials with immune properties. For example, Liu et al. designed a nanoparticle‐supported hybrid cytomembrane formulated with a fusion of cancer cells and DCs that not only express tumor antigens, but also potentiate immune stimulation for antitumor vaccination.^[^ ^214^ ^]^ Tumor antigen complexes and the immunological costimulatory molecules on cytomembranes could directly activate T cells or indirectly induce DC‐mediated T‐cell immune activation. Hybrid C6 glioma cell membranes and DC membranes were created to coat docetaxel (DTX) nanosuspensions.^[^ ^215^ ^]^ In 2021, Gong et al. constructed macrophages and 4T1 breast cancer cells hybrid‐biomimetic‐membrane‐camouflaged PLGA nanoparticles as a codelivery platform for immunometabolic modulator metformin (Met) and siRNA targeting fibrinogen‐like protein 1 mRNA (siFGL1) to possess the multitargeting capability.^[^ ^216^ ^]^

In addition, some membranes or vesicles with natural adjuvant activity can also be used to enhance immunotherapy. Cell‐derived nanovesicles (NVs) produced by serial sonication and extrusion of cell membranes are spherical membrane vesicles containing various biomolecules of cells and inherit multiple unique capabilities.^[^ ^235^ ^]^ Rao et al. reported a hybrid NVs containing PLT‐derived NVs, M1‐macrophage‐derived NVs, and cancer‐cell‐derived NVs overexpressing high‐affinity signal regulatory protein alpha (SIRP*α*) variants to accentuate macrophage phagocytosis of cancer cells, potentiate antitumor T‐cell immunity, and reduce side effects induced by systemic infusion.^[^ ^217^ ^]^ Chen et al. designed eukaryotic–prokaryotic‐vesicle (EPV)‐fused melanoma cytomembrane vesicles and attenuated *Salmonella* outer membrane vesicles (OMVs), which integrate tumor antigens with natural adjuvants for immunotherapy (Figure 16d).^[^ ^218^ ^]^ Wang et al. also constructed a hybrid membrane consisting of bacterial OMVs and B16F10 cancer cell membrane and coated it onto hollow polydopamine (HPDA) NPs.^[^ ^219^ ^]^ Liposomes with similar lipid bilayers of the membrane can also be fused with cell‐derived nanovesicles, compensating for their low yield, the complexity of surface functionalization, and heterogeneity in size. Hu et al. reported DOX‐loaded biomimetic hybrid nanovesicles (DOX@LINV) via fusing artificial liposomes with tumor‐derived nanovesicles (Figure 16e).^[^ ^220^ ^]^ DOX@LINV displayed an antitumor effect on murine melanoma, Lewis lung cancer, and 4T1 breast cancer. When combined with an immune checkpoint inhibitor, it amplified antitumor efficacy with 33.3% of the mice being tumor‐free. And recently, Hou et al. designed a hollow mesoporous Prussian blue (HMPB) nanosystem with mannose decoration and hydroxychloroquine (HCQ) adsorption, and then coated it with the hybrid macrophage and thylakoid (TK) membrane.^[^ ^221^ ^]^ TK–M@Man–HMPB/HCQ could suppress tumor growth via TAM polarization, CTL infiltration, hypoxia alleviation, and regulatory‐T‐cell decrement.

### 9.2 Protein‐Based Nanomaterials

**Figure 17:**
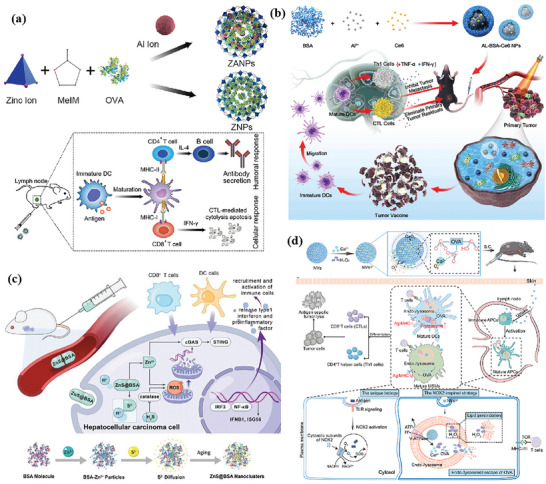
a) Synthetic aluminum‐integrated antigen–MOF (ZANPs) for effective targeting of lymph node (LN)‐resident DCs and efficient induction of immunotherapy. Reproduced with permission.^[^ ^63^ ^]^ Copyright 2019, Elsevier. b) Schematic illustration of Al–BSA–Ce6‐based immunotherapy. Reproduced with permission.^[^ ^222^ ^]^ Copyright 2020, Elsevier. c) The therapeutic process and synthesis routine of ZnS@BSA nanoclusters. Reproduced with permission.^[^ ^223^ ^]^ Copyright 2021, Wiley‐VCH. d) Schematic illustration of NVs^cp^ formation and the mechanisms of NVs^cp^‐induced cancer immunotherapy. Reproduced with permission.^[^ ^225^ ^]^ Copyright 2021, Elsevier.

Proteins have attracted considerable attention for their biocompatible and nontoxic characteristics. The unique spatial structure and molecular chain flexibility make protein a foundation for biomineralization.^[^ ^150^ ^]^ Minerals made by organisms are called biominerals, and the formation mechanisms are collectively termed biomineralization. As mentioned above, the protein–Fe–GA nanonetworks with intense near‐infrared absorption are based on a biomineralization strategy.^[^ ^150^ ^]^ Zhong et al. designed CpG/ZANPs synthesized by a natural biomineralization process with aluminum, OVA, and ZIF‐8. CpG was adsorbed on the surface of ZANPs to boost the immune response (**Figure** **17**a).^[^ ^63^ ^]^ BSA has been shown to chelate inorganic ions within nanoparticles under mild conditions.^[^ ^236^ ^]^ To integrate aluminum into nanoparticles, Zhu et al. engineered the photosensitizer Ce6 and immunoadjuvant aluminum ions into BSA (Al–BSA–Ce6 NPs) by albumin‐based biomineralization to achieve the synergy between PTT and cancer immunotherapy (Figure 17b).^[^ ^222^ ^]^ Cen et al. developed ZnS@BSA nanoclusters composed of BSA coloading zinc and sulfur via an ion diffusion approach for hepatocellular carcinoma (HCC) immunotherapy (Figure 17c).^[^ ^223^ ^]^ Albumin was selected for its strong affinity with metal ions, and zinc and sulfur ions were released under acidic TME to produce H_2_S that could specifically inhibit catalase activity in HCC cells. Su et al. devised biomineralized nanovaccines inspired by the nicotinamide adenine dinucleotide phosphate (NADPH) oxidase 2 mechanism, which were developed by in situ growth of calcium peroxide on nanovaccines self‐assembled with the model antigen OVA (Figure 17d).^[^ ^225^ ^]^ Calcium peroxide adjuvants responded to the acidic environment of the endo‐/lysosome and triggered the release of ROS to achieve endo‐/lysosomal escape. Another biomimetic strategy that fabricates hierarchical OVA@CaCO_3_ nanoparticles under the templating effect of antigen OVA was also developed.^[^ ^224^ ^]^ In addition, H_2_O_2_‐responsive manganese dioxide mineralized albumin (Alb) nanostructures loading with mitochondria function inhibitor phenformin and ICG were prepared by a two‐step biomineralization method for mild photothermal‐sensitized immunotherapy.^[^ ^226^ ^]^ Gu et al. described an antibody‐like polymeric nanoparticle composed of an albumin polymer hybrid nanoparticle core and an acid‐responsive PEG shell that can capture and remove galectin‐1 in tumor tissues to enhance the antitumor T‐cell responses.^[^ ^227^ ^]^

### 9.3 Nucleic‐Acid‐Based Nanomaterials

**Figure 18:**
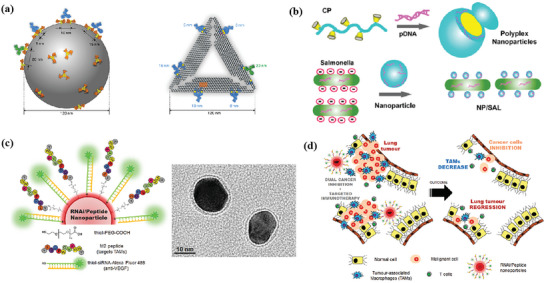
a) Schematic representation of noneven distribution of epitope spikes on the surface of a viral particle, and designed, virus‐mimicking DNA origami epitopes (DOEs) for IgG capture and binding. Reproduced with permission.^[^ ^229^ ^]^ Copyright 2020, Springer Nature. b) Engineering of polyplex‐nanoparticle‐coated *Salmonella*. Reproduced with permission.^[^ ^230^ ^]^ Copyright 2015, American Chemical Society. c) Gold nanoparticles functionalized with thiolated‐PEG—COOH conjugated to TAM‐targeting peptide (M2pep) and thiolated anti‐VEGF siRNA labeled with Alexa Fluor 488. d) Schematic of the outcome of the proposed combined silencing therapy (immunotherapy targeting TAMs and cancer cells) in vivo via highly specific and potent nanoparticles administered directly to bronchial airways. Reproduced with permission.^[^ ^233^ ^]^ Copyright 2015, Wiley‐VCH.

The application of DNA nanotechnology is rapidly expanding in the field of cancer immunotherapy. DNA can bind sequences specifically through the canonical pairing of its nucleobase substituents, which can be constructed as various complex structures such as DNA origami. Incorporating organic or inorganic molecules into DNA origami structures can improve stability and rigidity for in vivo applications.^[^ ^237^ ^]^ Liu et al. accurately hybridized an antigen peptide and two types of molecular adjuvants on the tubular DNA origami nanovaccine to stimulate tumor‐specific T‐cell responses that potently inhibit tumor growth.^[^ ^228^ ^]^ When localized in lysosomes in APCs, the nanodevice is triggered to mechanically expose antigens and adjuvants to activate a strong immune response due to low pH‐responsive DNA locking strands outside the nanostructures. Zhang et al. developed a triangular DNA origami framework, and six pairs of digoxin molecules were siting specifically anchored on the prescribed positions of the DNA origami to capture transient conformations of immunoglobulin Gs (IgGs) via atomic force microscopy (AFM), high‐speed AFM (HS‐AFM), and single‐molecule fluorescence resonance energy transfer (FRET) for virus neutralization, diagnostic detection, and cancer immunotherapy (**Figure** **18**a).^[^ ^229^ ^]^ HS‐AFM imaging of transient conformations provides structural and dynamic evidence for the IgG avidity from monovalent to bivalent in a single event, which reveals antibody–antigen interactions at the single‐molecule level and deepens the understanding of immunology. Otherwise, cationic polymers represent a promising type of nonviral gene delivery vectors and can be complex with nucleic acid electrostatic interactions. Hu et al. coated *Salmonella* with nanoparticles self‐assembled from cationic polymers PEI and DNA to overcome multiple barriers in the oral delivery of DNA vaccines (Figure 18b).^[^ ^230^ ^]^ The nanoparticles consisted of polymers and plasmid DNA could help facilitate bacteria escape phagosomes effectively and promote dissemination of bacteria into blood circulation. Moreover, DNA vaccines encoding autologous vascular endothelial growth factor receptor 2 showed remarkable T‐cell activation and cytokine production. CpG, the most potent immunostimulatory adjuvant for TLR9, is also a common nucleic acid. For example, Zhu et al. presented DNA inorganic hybrid nanovaccines that self‐assembled from concatemer CpG analogs and magnesium pyrophosphate (Mg_2_PPi).^[^ ^231^ ^]^ The inorganic Mg_2_PPi was dissolved in acidic endolysosomes of APCs, which helped CpG for efficient uptake into APCs and prolonged tumor retention to enhance cancer immunotherapy. Besides DNA, hybrid RNA materials are also the focus of cancer immunotherapy. To restore its immune activating efficacy in the hypoxic TME, Liu et al. encapsulated the lysine‐specific demethylase 1 (LSD1) siRNA and short dsRNA with a 5'‐triphosphate end (3pRNA) hybrid RNA structure in BSA‐coated MnO_2_ NPs.^[^ ^232^ ^]^ The hybrid RNA produces both endogenous retrovirus and exogenous viral signals for orchestrated immune activation. BSA‐coated MnO_2_ NPs efficiently relieve hypoxia, which awaken hypoxia‐mediated dormancy of viral RNA sensors and stimulates antitumor immunity. Otherwise, Conde et al. presented biohybrid RNA‐interference (RNAi)‐targeted peptide nanoparticles that can induce specific gene therapy in inflammatory TAMs in lung tumor tissue (Figure 18c,d).^[^ ^233^ ^]^ The targeting peptide and RNAi could transform tumor‐associated immune cells from an immunosuppressive to an immunostimulatory cell type by targeting the vascular endothelial growth factor (VEGF) pathway of M2 macrophages and cancer cells. In conclusion, the hybrid nucleic acids enable more control over nanometer spatial resolution, moving the field closer to real‐life properties and unprecedented biomedical applications.

## 10 Conclusion and Outlook

In recent years, hybrid nanomaterials have been developed and applied in various biomedical fields. Hybrid nanomaterials integrate the properties and functionalities of organic and inorganic materials, or diverse biomacromolecules, exhibiting good biocompatibility, biodegradability, drug loading capacity, and intrinsic activities in response to internal (intracellular environment, TME, etc.) or external stimuli (light, ultrasound, magnetic field, etc.). One typical example is hybrid organosilica nanomaterials, in which the incorporation of functional organic groups into the silica framework has turned such materials from bioinert to bioactive, resulting in improved responsive biodegradability, drug loading capacity, and immunoadjuvant activity. By taking advantage of the metal and organic moieties, MOFs and MPNs can intrinsically act as sensitizers to enable a range of therapeutic strategies, such as PTT, PDT, and RT for inducing the ICD effect, and/or serve as carriers for the delivery of therapeutics for eliciting systemic antitumor immunity. Generally, inorganic components play an important role in hybrid nanomaterials, but their biomedical applications are limited by poor biocompatibility and colloidal stability. The organic part in hybrid nanomaterials can at least partially overcome this drawback and introduce many other functions. For example, membrane‐based hybrid nanomaterials integrate the properties of multiple types of membranes, enabling the materials with improved circulation lifetime and biological targeting properties. Protein‐based hybrid nanomaterials are extensively used for their long half‐life, strong affinity with metal ions, and tumor‐targeting properties. Hybrid nucleic acid nanomaterials can be tuned with a wide range of architectures and can improve the stability of single DNA or RNA. As an emerging technology, it is expected that nucleic‐acid‐based covalent polymers and DNA origami will find more applications in the future. However, the application of biomacromolecules is usually limited because of the yield and high cost compared with other materials. Therefore, the approach of reducing the cost for large‐scale production in the future should be considered.

Despite significant progress that has been achieved, several issues still exist for the clinical translation of hybrid nanomaterials. First, the primary consideration of hybrid nanomaterials is biosafety. The multicomponent hybrid nanomaterials usually lead to complex biodegradation, metabolism, and excretion behaviors, particularly given the presence of metal ions as well as organic species that can be potentially toxic. Hence, systematic evaluations on the biocompatibility and biodegradability of hybrid nanomaterials in long term would be highly valuable. Second, TME is composed of cancer cells, vascular endothelial cells, and tumor‐infiltrating immunocytes, etc., which contributes to immunosuppressive and decreases the efficacy of hybrid nanoparticles. The immunosuppressive mechanisms of TME and the transport and degradation of hybrid nanoparticles in TME need to be considered carefully in different tumor types. Particularly, in order to ensure biological safety and reduce the possible toxicity of the nanoparticles, the appropriate administration routes need to be selected in antitumor therapy. Third, the relatively complex composition of hybrid nanomaterials poses difficulty for large‐scale production and reproducibility. Therefore, developing simple, versatile, scalable, and reproducible synthetic strategies for hybrid nanomaterials are vital for improving their clinic potential. Finally, as comprehensively discussed in this review, the large proportion of studies relating to hybrid‐nanomaterial‐mediated cancer immunotherapy involves other therapeutic modalities, for instance, PDT, PTT, RT, and chemotherapy, and in many cases, more than two therapeutic modalities are involved. Whereas those combinational strategies showed impressive immunotherapeutic outcomes, their clinic translation can be challenging due primarily to the complexity of constructing those nanosystems, optimizing the dosage and timing of each therapeutic modality, understanding their interaction with various biotargets, and controlling the adverse effects. Therefore, we do recommend that both the composition of hybrid nanomaterials and the components of the nanosystems should be kept as simple as possible under the premise that the ultimate therapeutic efficacy is not compromised.

We envision that, in the near future, there are a number of promising areas of hybrid nanomaterials that are worth to be further investigated in cancer immunotherapy. First, for PTT, some metal carriers like gold nanoparticles (GNPs) have photothermal conversion properties. Due to the enhanced permeability and retention effect, GNPs can be injected intravenously and accumulate preferentially in cancer cells.^[^ ^238^ ^]^ Among GNP platforms, gold nanostars have great therapeutic potential due to their unique star‐shaped geometry that dramatically enhances light absorption and provides high photon‐to‐heat conversion efficiency due to the plasmonic effect.^[^ ^239^ ^]^ This photothermal process can be exploited to specifically ablate tumors and, more importantly, to amplify the antitumor immune response following the highly immunogenic thermal death of cancer cells.^[^ ^238^ ^]^ However, there are no reported examples of combining metal carriers with photothermal conversion properties with hybrid nanomaterials, which is already a widely used means in non‐immunotherapy. Therefore, the combination of metal carriers with PTT effect and immunotherapy will also be one of the directions of future exploration in the field of hybrid‐nanomaterial‐based immunotherapy. In addition, mammalian viruses have been used to improve conventional cancer therapy, especially for immunotherapy. For instance, oncolytic viruses (OVs) are capable of exerting anticancer effects by targeting and killing tumor cells, which can also induce the immune response by combining tumor lysis with induction of typical antiviral responses.^[^ ^240^ ^]^ In contrast with OVs, plant virus nanoparticles as a novel immunostimulatory agents, have better biocompatibility, biodegradability, and potential clinical value. However, the combination of viruses and organic/inorganic nanomaterials for cancer immunotherapy is rarely reported to stimulate an antitumor immune response, which can be further studied. Besides viruses, it is worth mentioning that mRNA has gained tremendous popularity in cancer immunotherapy compared to conventional vaccines currently.^[^ ^241^ ^]^ Hybrid nanomaterials can be used for the delivery of mRNA vaccines as novel vectors to overcome the instability and the delivery of mRNA. Finally, CAR T‐cell therapy interceding antitumor effects by transfusing T lymphocytes has been approved for the treatment of patients with malignancies.^[^ ^242^ ^]^ CAR‐T cells loaded with hybrid nanoparticles rather than traditional nanoparticles may endow more function when delivering cargo to tumor site and lymph node efficiently, stimulating the expansion of CAR‐T cells. CAR natural killer (NK) and CAR macrophage therapy can also be combined with hybrid nanomaterials for cancer immunotherapy as an alternative approach for adoptive cell therapy that are actively explored.^[^ ^243^ ^]^ In conclusion, hybrid nanomaterials for cancer immunotherapy are in the infant stage and few have entered into clinical stage. More efforts are needed to elucidate immunological behavior as well as biosafety profile of hybrid nanomaterials with sophisticated compositions, for the sake of improving their translational potential.

## Conflict of Interest

The authors declare no conflict of interest.
